# Marine Sulfated Polysaccharides as Promising Antiviral Agents: A Comprehensive Report and Modeling Study Focusing on SARS CoV-2

**DOI:** 10.3390/md19080406

**Published:** 2021-07-22

**Authors:** Abdalla E. M. Salih, Bathini Thissera, Mohammed Yaseen, Ahmed S. I. Hassane, Hesham R. El-Seedi, Ahmed M. Sayed, Mostafa E. Rateb

**Affiliations:** 1 School of Computing, Engineering & Physical Sciences, University of the West of Scotland, Paisley PA1 2BE, UK; b00382049@studentmail.uws.ac.uk (A.E.M.S.); Bathini.Thissera@uws.ac.uk (B.T.); Mohammed.Yaseen@uws.ac.uk (M.Y.); ahmedsayed.hassane@nhs.scot (A.S.I.H.); 2 Aberdeen Royal Infirmary, Foresterhill Health Campus, Aberdeen AB25 2ZN, UK; 3 Pharmacognosy Group, Department of Pharmaceutical Biosciences, BMC, Uppsala University, Uppsala, Box 591, SE 751 24 Uppsala, Sweden; hesham.el-seedi@farmbio.uu.se; 4 International Research Center for Food Nutrition and Safety, Jiangsu University, Zhenjiang 212013, China; 5 Department of Chemistry, Faculty of Science, Menoufia University, Shebin El-Kom 32512, Egypt; 6 Department of Pharmacognosy, Faculty of Pharmacy, Nahda University, Beni-Suef 62513, Egypt

**Keywords:** sulfated polysaccharides, antiviral, SARS-CoV-2, docking, molecular dynamic simulations

## Abstract

SARS-CoV-2 (severe acute respiratory syndrome coronavirus-2) is a novel coronavirus strain that emerged at the end of 2019, causing millions of deaths so far. Despite enormous efforts being made through various drug discovery campaigns, there is still a desperate need for treatments with high efficacy and selectivity. Recently, marine sulfated polysaccharides (MSPs) have earned significant attention and are widely examined against many viral infections. This article attempted to produce a comprehensive report about MSPs from different marine sources alongside their antiviral effects against various viral species covering the last 25 years of research articles. Additionally, these reported MSPs were subjected to molecular docking and dynamic simulation experiments to ascertain potential interactions with both the receptor-binding domain (RBD) of SARS CoV-2’s spike protein (S-protein) and human angiotensin-converting enzyme-2 (ACE2). The possible binding sites on both S-protein’s RBD and ACE2 were determined based on how they bind to heparin, which has been reported to exhibit significant antiviral activity against SARS CoV-2 through binding to RBD, preventing the virus from affecting ACE2. Moreover, our modeling results illustrate that heparin can also bind to and block ACE2, acting as a competitor and protective agent against SARS CoV-2 infection. Nine of the investigated MSPs candidates exhibited promising results, taking into consideration the newly emerged SARS CoV-2 variants, of which five were not previously reported to exert antiviral activity against SARS CoV-2, including sulfated galactofucan (**1**), sulfated polymannuroguluronate (SPMG) (**2**), sulfated mannan (**3**), sulfated heterorhamnan (**8**), and chondroitin sulfate E (CS-E) (**9**). These results shed light on the importance of sulfated polysaccharides as potential SARS-CoV-2 inhibitors.

## 1. Introduction

Mother Nature remains an outstanding hub for valuable natural compounds that have been used for multiple purposes since ancient times by our ancestors, including pharmaceutical, biomedical, nutritional, and cosmetics applications. Among the dozens of natural product groups, polysaccharides are macromolecular polymeric carbohydrate molecules comprised of large chains of monosaccharide units. They are widely found in animals, plants, and microorganisms, and their functions are mainly either structure- or storage-related. Many studies have revealed that natural polysaccharides and their chemically modified derivatives have significant inhibitory activities against viral diseases such as human immunodeficiency virus (HIV) and herpes simplex virus (HSV) [1,2].

Sulfated polysaccharides (SPs) are a class of negatively charged polysaccharides comprising natural or modified sulfate moieties in their structural carbohydrate backbone. They possess significant biological activities such as antioxidant, anti-allergic, antiviral, anticancer, and anticoagulant abilities; hence, the study of SPs has significant importance for drug discovery campaigns [1,3]. SPs are mainly found in the cell walls of marine algae or seaweeds; they are less common in some mammals, such as fish skins, and rare in mangrove plants. The seaweed cell wall comprises about 40% of sulfated polysaccharides, which is relatively higher than the average content in other sources. The most interesting marine algal SPs are sourced from ulvans from green macroalgae, carrageenans and agar from red macroalgae, and fucoidans and laminarians from brown macroalgae. These SPs have shown antiviral activity against herpes simplex virus (HSV), human immunodeficiency virus type-1 (HIV-1), chikungunya virus, cytomegalovirus (CMV), influenza virus, and hepatitis virus, in addition to other enveloped and non-enveloped viruses. Moreover, the antiviral activity of SPs against the current COVID-19 pandemic has also been reported [1,4,5].

Marine sulfated polysaccharides (MSPs) have recently received increasing attention due to their antiviral activity. In particular, carrageenans have exhibited promising inhibitory effects on many viral strains, effectively preventing the internalization of virus particles by interfering with the interactions between the virus and host cell receptors. Carrageenan nasal spray (Boots Dual Defence^®^ in the UK market) has been proven effective in patients with common cold infected by human coronaviruses beta and alpha. In contrast, the impact was attributed to the increased viral clearance and the reduced relapses of symptoms in children and adults [6]. Another nasal spray formulation (xylometazoline HCl) containing iota-carrageenan (**4**) can effectively relieve nasal congestion of the upper respiratory tract and protect the respiratory mucosa against viral infection [7]. Coldamaris^®^ lozenges, comprising iota-carrageenan (**4**) as the active pharmaceutical ingredient, cause denaturation of glycoproteins on the coronavirus surface, inhibiting the virucidal effects of coronavirus [8].

It remains challenging to develop novel drugs within a limited timeframe, as drug discovery is time- and resource-consuming; however, the process of drug discovery is immensely enhanced by the modern era of computational technology, which has accelerated drug discovery and drug repurposing. Computer-assisted or in silico design employs computational methods in drug discovery and is being applied to streamline and speed up hit-to-lead optimization and hit identification. Several computational techniques have been introduced to predict and select therapeutic targets, study the interactions between drug and receptor, characterize and determine ligand binding sites on the targets, and determine hit compounds using ligand- and structure-based virtual screening [9,10].

Molecular docking and dynamic simulations are the most intriguing among the computational tools employed for structure-based drug discovery, an approach that evaluates the binding affinities between two candidates: small molecules and macromolecular targets (protein). Many drugs currently in the market were developed based on in silico strategies, such as zanamivir (used to treat influenza), nelfinavir, and saquinavir (used in the treatment of HIV); therefore, computational methods have been receiving popularity in the pharmaceutical industry as being crucial in the drug discovery process as reliable and effective techniques [11,12,13].

In the present investigation, we aimed to shed light on the antiviral potential of MSPs, illustrating their modes of interaction against different targets with the aid of several in silico tools, highlighting the most potential candidates for further investigation against SARS CoV-2.

## 2. Results and Discussion

**Figure 1:**
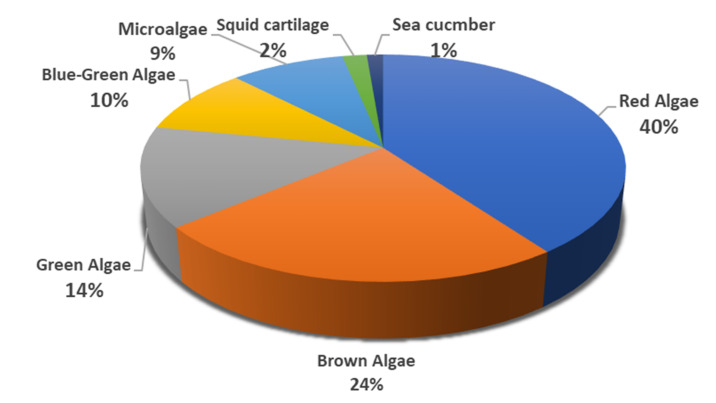
The systematic study of marine sources of MSPs that have shown antiviral activities in the last 25 years.

Herein, we have summarized the findings of approximately 80 research studies published in the last 25 years on MSPs that exhibited potential antiviral effects against a total of 22 different viral strains (Table 1, Figure S1). These antiviral MSPs were sourced from various marine sources, including brown algae, red algae, green algae, blue-green algae, microalgae, sea cucumber, and squid cartilage. According to the findings, 40% of the antiviral MSPs were isolated from the red algae, followed by 24% from brown algae, 14% from green algae, 10% from blue-green algae, 9% from microalgae, 2% from squid cartilage, and 1% from sea cucumber (Figure 1). The red-algae-derived MSPs exhibited greater efficacy against several viruses; however, the distribution was highly skewed towards herpes simplex viruses (HSV), which received most of the research effort (Figure S1).

### 2.1. MSPs from Red Algae

Red macroalgae were commercially considered more valuable than brown and green macroalgae and are widely used in manufacturing hydrocolloids, such as carrageenan and agar, which in turn are involved many applications, such as food, pharmaceutical, and biotechnological industries. The principal polysaccharide components of the red algae are sulfated galactans that are produced extracellularly; however, they are made up of a linear backbone of alternating 3-linked β-D-galactopyranose and 4-linked α-D-galactopyranose, with a few exceptions, such as DL-hybrid sulfated galactan. The various structural types of sulfated galactan (**7**) have revealed potent antiviral activity against several types of enveloped viruses, such as HSV-1, HSV-2, DENV-2, HIV-1, and HIV-2 (Table 1).

**Figure 2:**
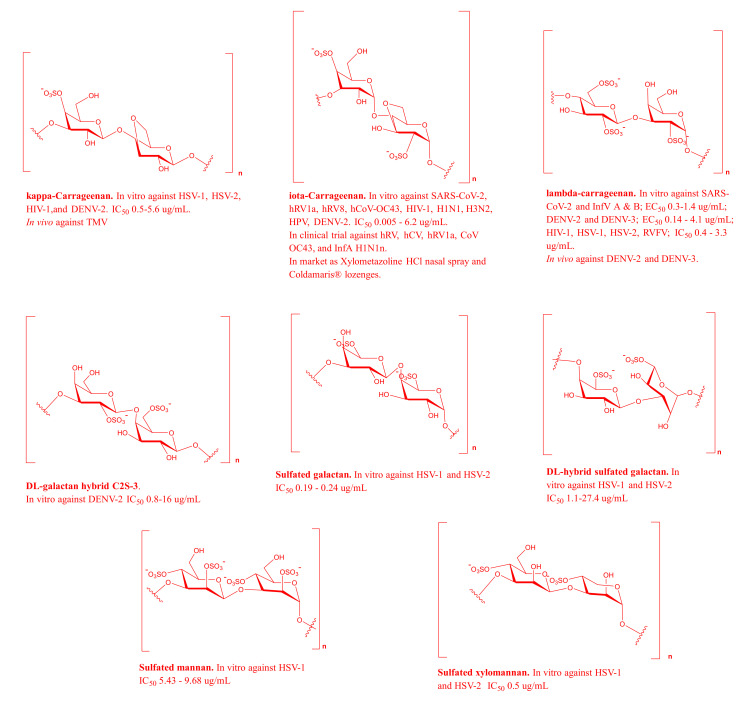
Representative MSPs from red algae.

Carrageenans, which are isolated from the cell walls, represent about 30–70% of the red algal dry weight, and their structure consists of linear chains of alternating galactopyranose units with linkages between 1 and 3 monomeric positions and other galactopyranose units with linkages between 1 and 4 monomeric positions or 3,6-galactopyranose units. The most important and extensively studied carrageenans are kappa (κ), iota, and lambda (λ), which vary in the number and position of sulfate ester groups (S), in accordance with the presence of 3,6-anhydrous-D-galactopyranose units [4]. Carrageenans are broad-spectrum antivirals and have recently shown significant inhibitory effects by preventing the physical binding and entry of viral particles. Carrageenans were found to be effective against 12 different viruses (HSV, SARS-CoV-2, InfV, hRV, hCoV-OC43, HIV, DENV, hCV, HPV, RVFV, JEV, and TMV). Additionally, carrageenans are the most investigated class in human clinical trials against various virus diseases, such as sexually transmitted HIV, HPV, and HSV, in addition to rhinoviruses [101]. The most successful antiviral preparations of carrageenans were the nasal spray dosage forms that have recently been developed against rhinoviruses and SARS-CoV-2 [6,7,18,43]. Alongside carrageenan and sulfated galactans, other MSPs extracted from red algae and showed considerable activities against HSV, such as sulfated xylomannans, sulfated mannan, sulfated xylogalac tans, and sulfated xylan (Table 1, Figure 2).

### 2.2. MSPs from Brown Algae

**Figure 3:**
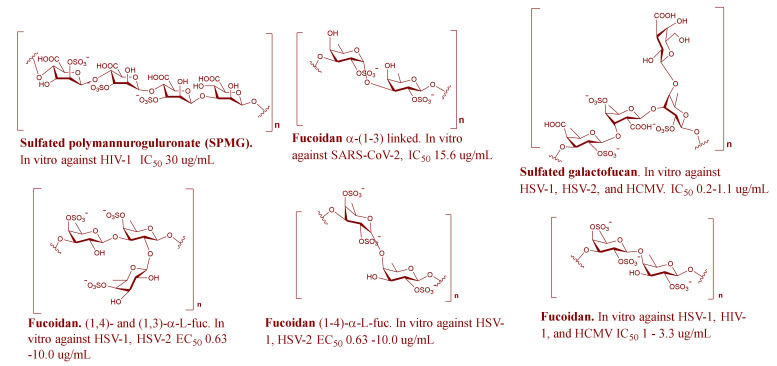
Representative MSPs from brown algae.

Brown algae come in second place after red algae in terms of antiviral activities and research attention (Figure 1). The main reported MSPs from this genus were fucoidans, a group of heterogeneous sulfated polysaccharides that represent about 25–30% of the algae dry weight and are made up of a backbone of α-(1→3)-L-fucopyranose residues or alternating α-(1→3) and α-(1→4)-linked L-fucopyranosyls with a sulfate group mainly substituted on C-2 or C-4 fucopyranose residues [4]. Fucoidans were found to be effective against 7 different viruses (HSV, SARS-CoV-2, InfAV, HIV-1, DENV-2, NDV, and HCMV) (Table 1, Figure 3 and Figure S1).

Generally, fucoidans block viral infection by preventing viral entry through competing for the positive charge attachment site of the envelope glycoproteins. The extent of antiviral activity is related to the number of sulfate groups present in the fucoidan structure [102]. As illustrated in Table 1, fucoidans have been tested in vivo using mice or mouse models for their activity against InfAV and HSV-2 and showed promising results. A recent in vitro study of two different fucoidans revealed that these compounds could be potent inhibitors of SARS-CoV-2 [17,18].

### 2.3. MSPs from Green Algae

**Figure 4:**
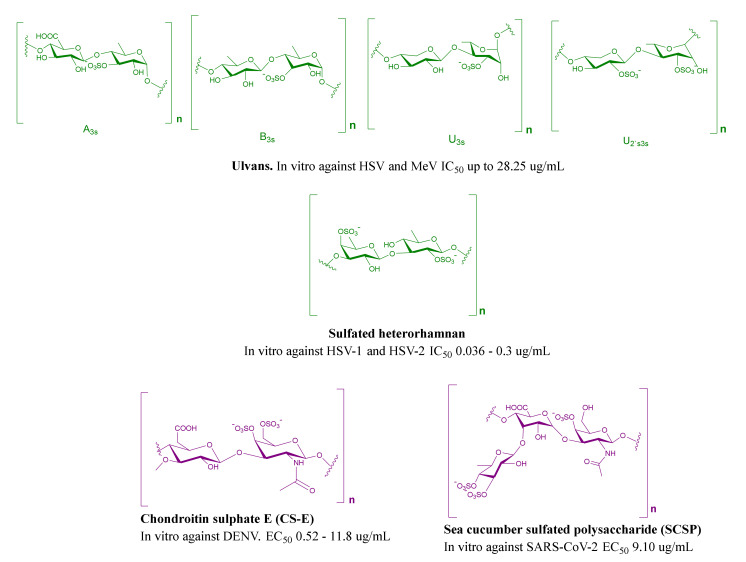
Representative MSPs from green algae (green structures), blue-green algae, and marine-animal-derived SPs (violet structures).

Green-algae-derived MSPs come in third place as the most-investigated algae. Most of the examined compounds for antiviral activities belong to Ulvanes (Monostroma, Ulva, Enteromorpha), *Codium*, and *Caulerpa*. Different green-algae-derived MSPs were studied in vitro and in vivo against 11 viruses, including avian InfAV, HSV, HCMV, DENV-2, EV71, MeV, NDV, MuV, hCV, HIV, and JEV (Table 1, Figure 4). Song and his colleagues examined the antiviral activity of *Ulva pertusa* against avian InfAV and found that it exhibited a mild antiviral effect (40% viral inhibition). When combined with a vaccine against the same virus, it generated a synergistic effect, which significantly enhanced the production of antibodies by more than two-fold (~100%) [86]. Although the antiviral activities of green algae have received less attention than the red and brown, they possess unique antiviral properties against diverse viruses, such as NDV [78] and JEV [85].

### 2.4. MSPs from Miscellaneous Marine Sources

Blue-green algae, microalgae, sea cucumbers, and squid cartilage (Table 1, Figure 4) are studied much less than the former algae for their MSPs as potential antiviral agents. Among them, the novel sulfated polysaccharide calcium spirulan (Ca-SP) was isolated from the blue-green alga *Spirulina platensis* and found to be an inhibitor for several viruses, including HSV-1, HCMV, InfAV, MeV, HIV-1, and Muv [88]. Recently, a clinical trial confirmed the potential of Ca-SP (Spirularin^®^ HS) against herpes viruses through inhibiting the attachment and penetration of HSV-1 into mammalian epithelial cells and blocking the entry of Kaposi sarcoma-associated herpesvirus HSV-8 [90]. The highly sulfated polysaccharide p-KG03 isolated from the microalgae *Gyrodinium impudium* showed potent inhibitory activity against InfAV by blocking the early stage of replication and entry [95]. Sea cucumber sulfated polysaccharide (SCSP) is one of the most recent MSPs extracted from the sea cucumber *Stichopus japonicus*. Song and colleagues confirmed its activity against SARS-CoV-2 in an in vitro study through binding to the S-glycoprotein, preventing SARS-CoV-2 host cell entry [18].

### 2.5. In Silico Investigation of MSPs against SARS CoV-2

**Figure 5:**
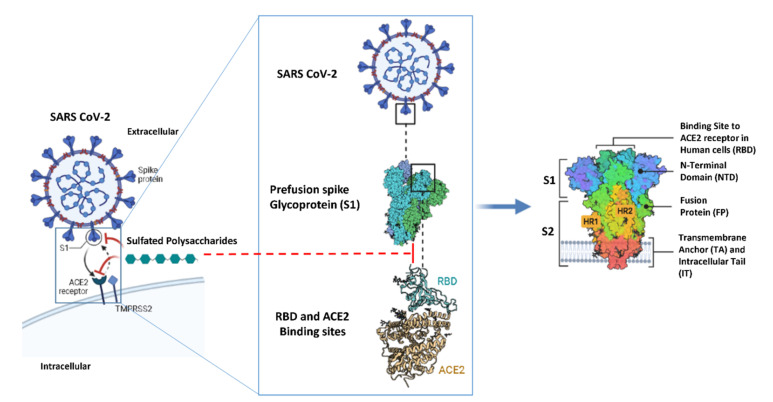
Schematic representation of the structure of SARS CoV-2 S-protein and how it can bind to the human ACE2. (PDB code: 6VXX) [104].

Molecular modeling of polysaccharides is not an easy task due to their high diversity, complexity, and flexibility; however, the recent advances with in silico tools can relieve the complexity of the process to a greater extent. Recently, heparin (**10**), an example of sulfated polysaccharide, was reported to exhibit a promising antiviral activity against SARS CoV-2 (EC_50_ = 36 µg/mL) through S-protein binding; hence, it suppresses the viral attachment to ACE2 (SARS CoV-2 S-protein receptor) and subsequently its entry inside the host cell (Figure 5) [17,103]. Similarly, most of the reported MSPs were found to mediate their antiviral activity via the exact mechanism, particularly those reported as anti-SARS CoV-2.

**Figure 6:**
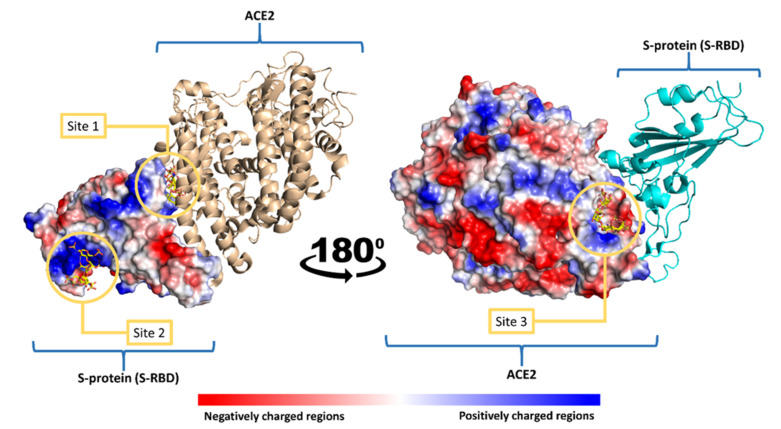
SARS CoV-2′s S-RBD–ACE2 complex showing heparin binding sites (sites 1, 2 and 3).

Accordingly, in this study, we shed light on MSPs as potential SARS CoV-2 antiviral agents by speculating on their plausible mode of action at the molecular level using a series of molecular docking and dynamic simulation experiments. Firstly, we determined the possible binding sites on both the S-protein receptor-binding domain (S-RBD) and ACE2 for MSPs. To do so, we utilized ClusPro [105], a software specialized in the prediction of heparin (**10**) (i.e., as an example of sulfated polysaccharide) binding sites in any given protein via molecular docking. The predicted heparin-S-RBD and heparin-ACE2 complexes were then subjected to 50 ns molecular dynamic simulation (MDS) experiments to select the most stable binding modes with each protein (S-RBD and ACE2). As shown in Figure 6, heparin was predicted to achieve stable binding with S-RBD at two sites (sites 1 and 2). Site 1 is located in a region that can interact with ACE2 directly and has a moderate positive charge. In contrast, site 2 is shallower than site 1 and is located in a wide positively charged region. Heparin-S-RBD complexes in these two sites were significantly stable over the course of MDS with low deviations from the original poses (RMSD ~ 2.5 Å) and minimal fluctuations (site 1 and site 2, Figure 6). This apparent stability resulted from the networks of H-bonds and ionic interactions formed between heparin and each binding site (Table 2 and Table 3).

On the other hand, heparin was predicted to interact with a small pocket (site 3) on ACE2 located near the S-RBD binding region (Figure 6). Similar to sites 1 and 2, heparin achieved stable binding with site 3 during the MDS (RMSD ~ 1.9 Å) through extensive H-bonds and ionic interactions (site 3, Table 4 and Figure 6).

**Figure 7:**
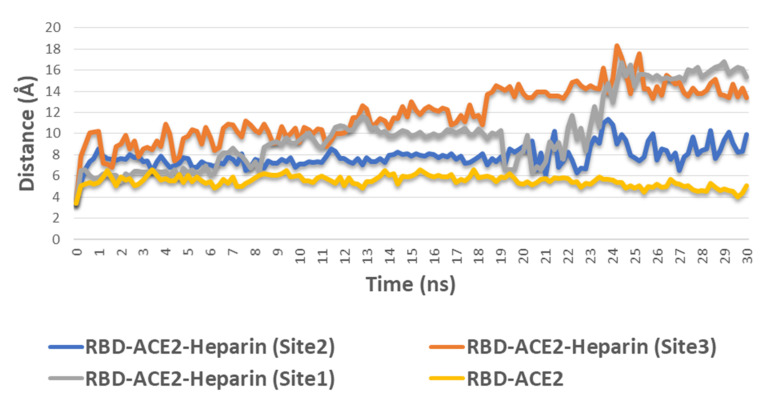
The calculated distance between S-RBD and ACE2 (i.e., between GLN-493 and GLU-35, respectively) during 30 ns of MDS in the absence and presence of heparin in site 1, 2, or 3.

The complex stability of RDB–ACE2 was also studied upon heparin binding to each site. As depicted in Figure 4, the distance between S-RBD and ACE2 (calculated as the distance between GLN-493 and GLU-35, respectively) remained constant (~5.1 Å) over 30 ns of MDS, while binding of heparin to S-RBD or ACE2 via sites 1, 2, or 3 led to significant instability of the complex and gradual dissociation (i.e., increased distance between GLN-493 and GLU-35). Heparin binding to site 1 on S-RBD or site 3 on ACE2 showed the greatest effects as S-RBD dissociated almost completely from ACE2 after 20 ns of simulation (Figure 7). Accordingly, it can be concluded that stable binding of SPs to sites 1, 2, or 3 destabilizes the S-RBD–ACE2 complex.

**Figure 8:**
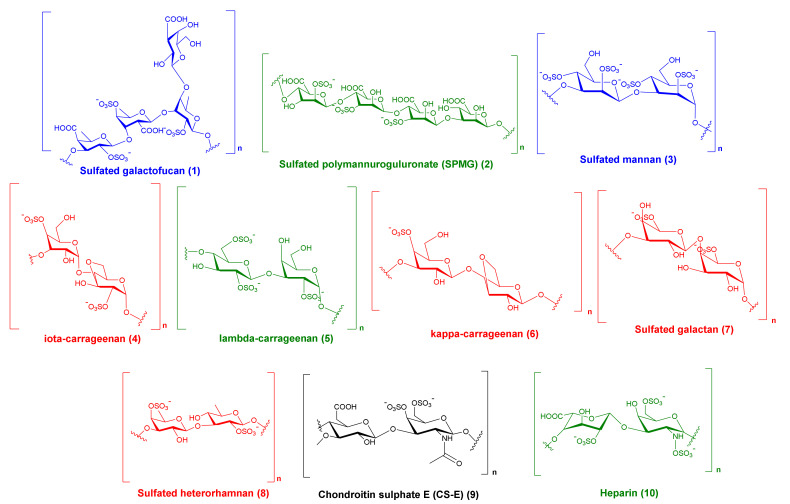
Sulphated polysaccharides with docking scores <−5 kcal/mol. Red compounds were significantly unstable during the course of MDS experiments. Black compound (compound 9) achieved stable binding with site 1 only. Blue compounds achieved stable bindings with sites 1 and 2. Green compounds achieved stable bindings with sites 1, 2, and 3.

It was recently reported that heparin can bind to S-RBD, preventing SARS CoV-2 from reaching ACE2 and entering the host cell; however, according to our modeling results, heparin can also bind to and block ACE2, acting as a protective agent against SARS CoV-2 infection. To study the mode of action of all reported MSPs against SARS CoV-2 to suggest previously unreported candidates, we subjected all collected compounds (Table 1) to dock against the proposed binding sites (sites 1, 2, and 3). Top-scoring hits (Figure 8) were selected according to the following criteria: (i) Docking score < −5 kcal/mol. Scores >−5 (i.e., −4.9 to −1.2 kcal/mol) showed unstable binding with the corresponding protein (RMSD > 20 Å at the first 20 ns); (ii) ΔG value < −5 kcal/mol, (iii) the docking pose remains stable over 50 ns of MDS. Some compounds achieved docking scores <−5 kcal mol (e.g., −6.4 kcal mol), although they were significantly unstable during the MDS experiments (RMSD > 15 Å at the first 20 ns) and presented significantly higher ΔG values (~−1.3 kcal/mol). As such, we made the binding stability at least 50 ns alongside ΔG value < −5 kcal/mol another selection criterion to discriminate between binders from non-binders, ensuring that all selected hits can achieve stable binding with the corresponding protein.

Additionally, we took into consideration the newly emerged SARS CoV-2 variants during our docking experiments. Upon reviewing the recent mutations of the viral S-protein, we found that the UK variant (i.e., B.1.1.7) has a mutation in its S-RBD (i.e., N501Y) that was associated with its higher affinity to the ACE2 receptor [106]. This mutation was the replacement of the amino acid ASP-501 with TYR. We applied this mutation to the S-RBD structure to study its effect on the binding with MSPs upon docking and MDS. 

As shown in Table 2, docking against site 1 (with and without the N501Y mutation) resulted in nine SPs with scores <−5 kcal/mol (Figure 8). Four of these hits were previously reported to exert antiviral activity against SARS CoV-2. Further MDS and ΔG experiments revealed that the best binding modes for iota-carrageenan (**4**), kappa-carrageenan (**6**), sulfated galactan (**7**), and sulfated heterorhamnan (**8**) (Figure 8) with site 1 were significantly unstable (their average RMSD values were higher than 15 Å and their ΔG values were higher than −5 kcal/mol) and they could easily dissociate from site 1 (with and without the N501Y mutation). Sulfated galactofucan (**1**), sulfated mannan (**3**), and chondroitin sulphate E (CS-E) (**9**) were among the best-scoring hits that were significantly stable during the MDS experiments, either with the mutated or non-mutated RBD. Additionally, they had not been previously reported for their antiviral efficacy against SARS CoV-2; hence, they were considered good candidates for future evaluation.

**Figure 9:**
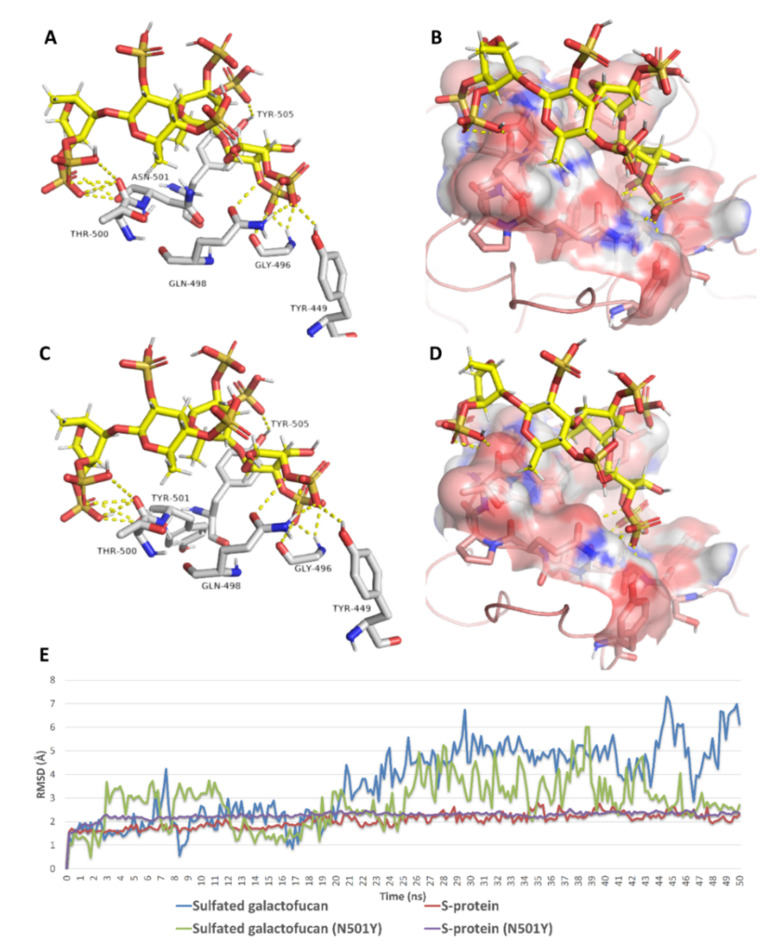
Binding mode of sulphated galactofucan (compound **1**) inside site 1 (**A**,**B**) and its mutated form (**C**,**D**) together with its RMSDs during 50 ns of MDSs (**E**).

**Figure 10:**
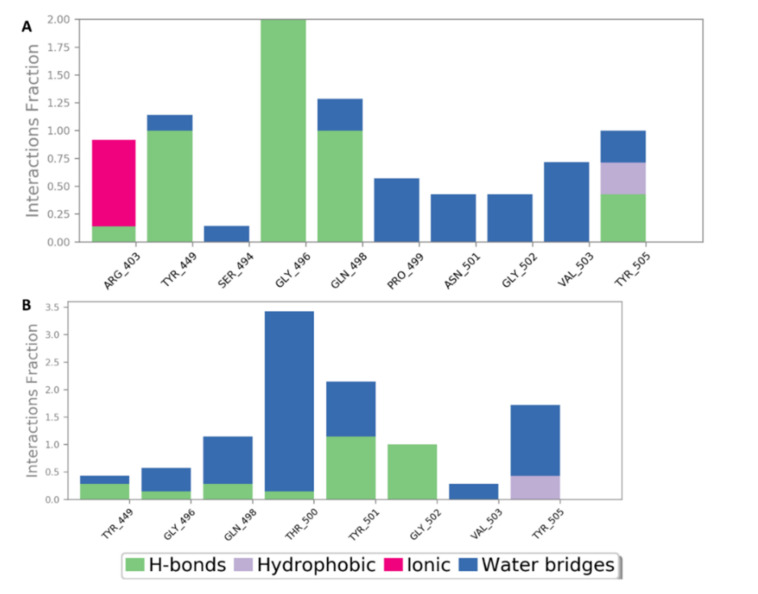
Protein–ligand contacts of sulphated galactofucan (compound 1) inside site 1 (**A**) and its mutated form (**B**) during the MDS.

**Figure 11:**
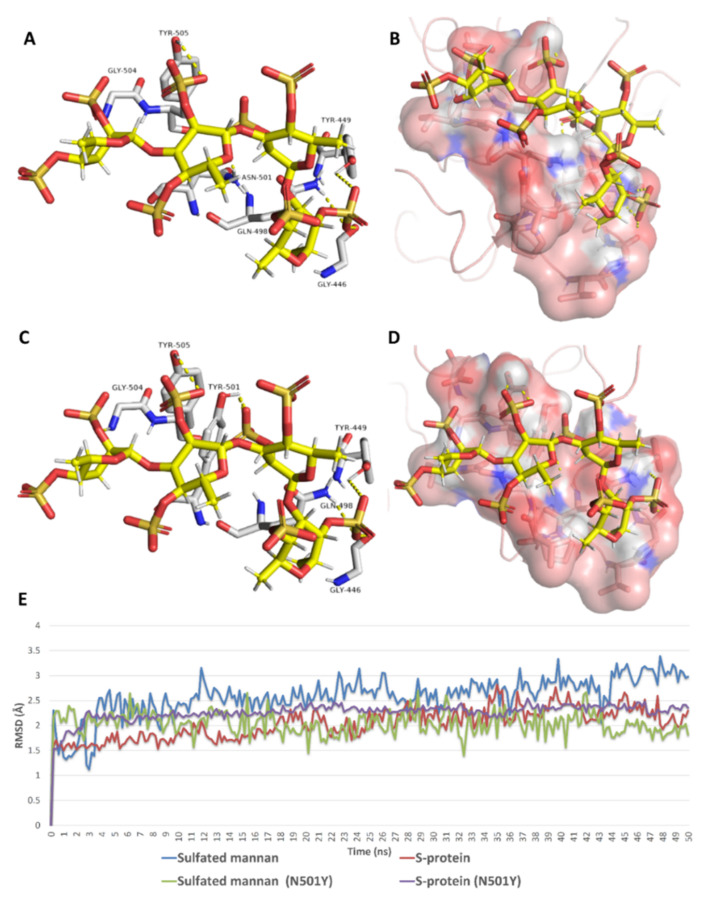
Binding mode of sulfated mannan (**3**) inside site 1 (**A**,**B**) and its mutated form (**C**,**D**) together with its RMSDs during 50 ns of MDSs (**E**).

**Figure 12:**
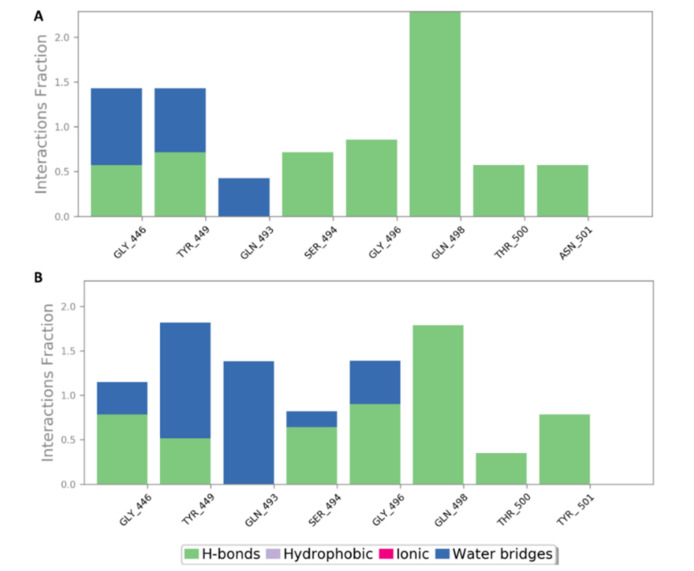
Protein–ligand contacts of sulfated mannan (**3**) inside site 1 (**A**) and its mutated form (**B**) during the MDS.

**Figure 13:**
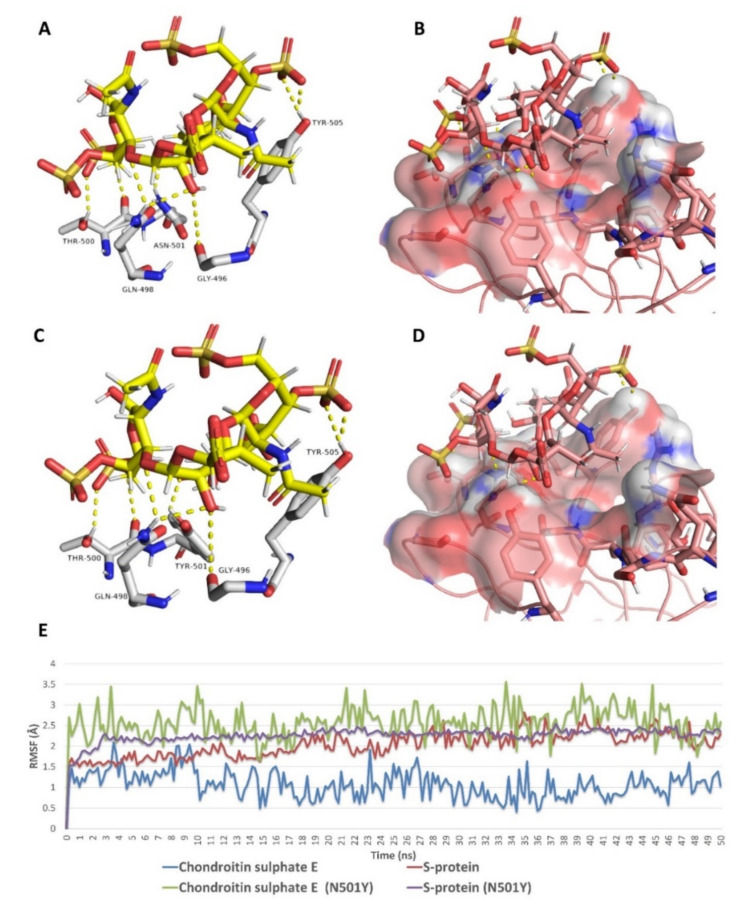
Binding mode of chondroitin sulphate E (compound **9**) inside site 1 (**A**,**B**) and its mutated form (**C**,**D**) together with its RMSDs during 50 ns of MDSs (**E**).

**Figure 14:**
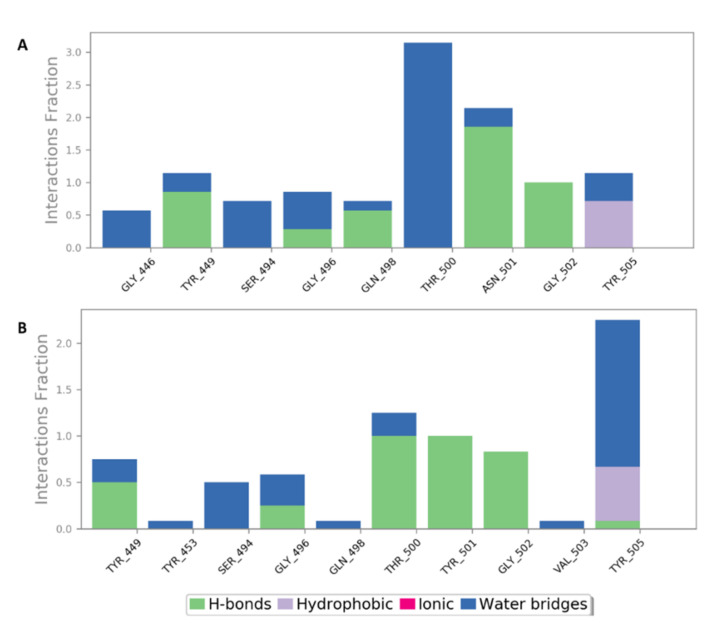
Protein–ligand contacts of chondroitin sulphate E (compound 9) inside site 1 (**A**) and its mutated form (**B**) during the MDS.

As shown in Figure 9, Figure 10, Figure 11, Figure 12, Figure 13 and Figure 14, the prevalent interactions of these MSPs and RBD site 1 in its mutated and non-mutated forms were H-bonds and water bridges, where the sulfate esters were the main contributors in these interactions with site 1 key amino acid residues (Table 2). Moreover, their fluctuations and deviations from the starting docking poses were almost identical to both the mutated and non-mutated form of site 1 during the MDS, except for chondroitin sulphate E, which achieved slightly more stability and less deviation from the starting binding pose upon binding to the non-mutated form of site 1 (Figure 13).

**Figure 15:**
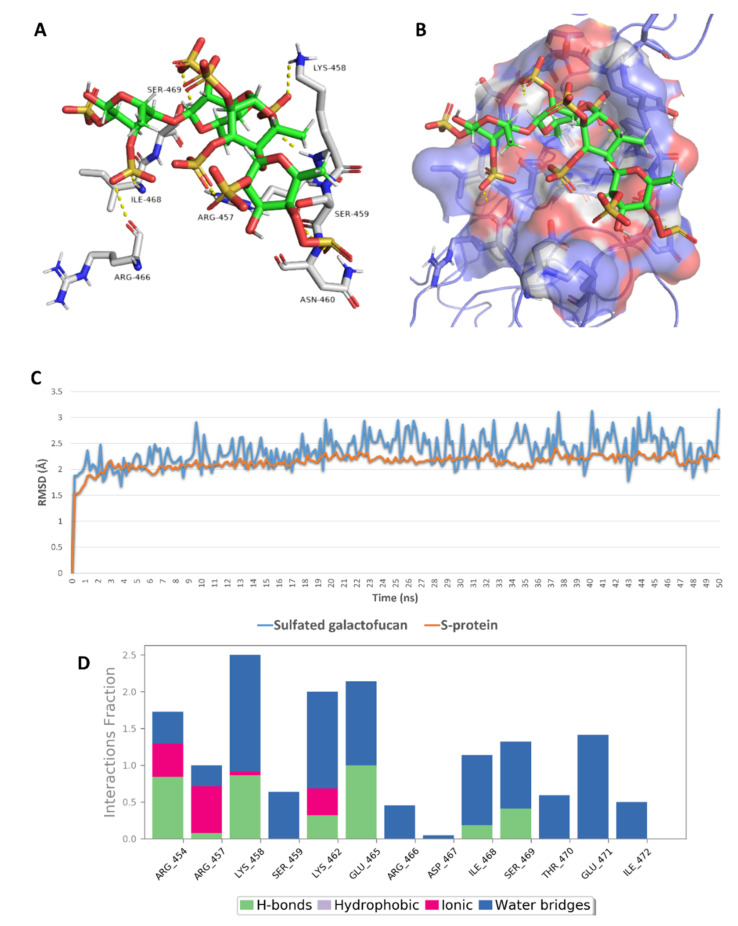
Binding mode of sulfated galactofucan (**1**) inside site 2 (**A**,**B**) together with its RMSDs and protein–ligand contacts during 50 ns of MDSs (**C**,**D**).

**Figure 16:**
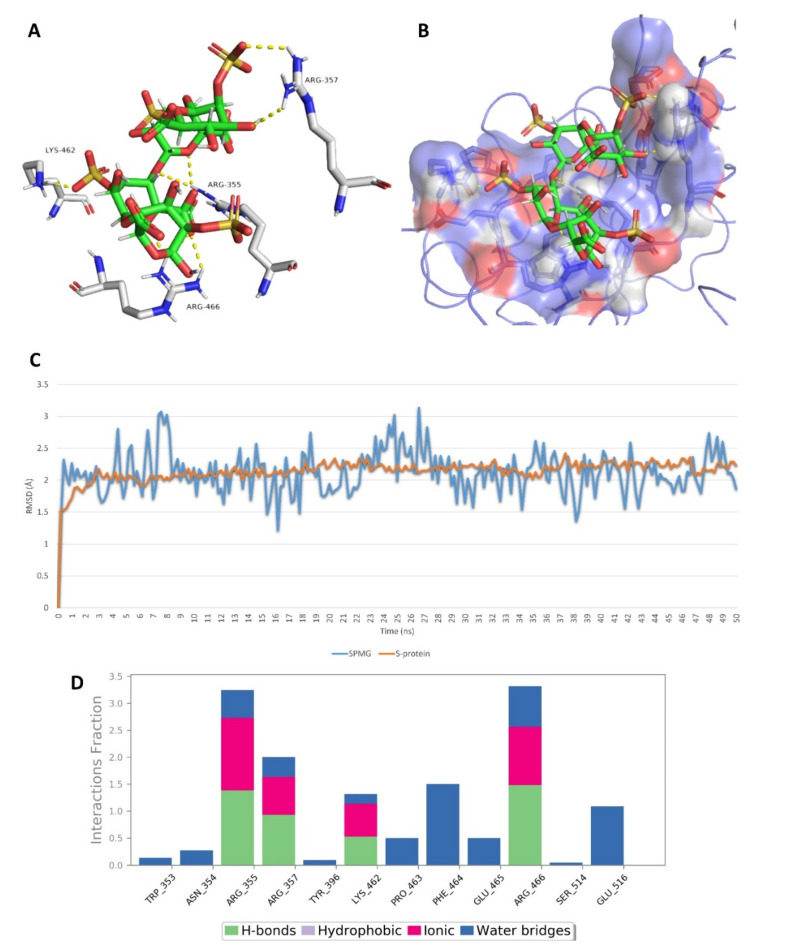
Binding mode of sulfated polymannuroguluronate (SPMG) (compound 2) inside site 2 (**A**,**B**) together with its RMSDs and protein–ligand contacts during 50 ns of MDSs (**C**,**D**).

**Figure 17:**
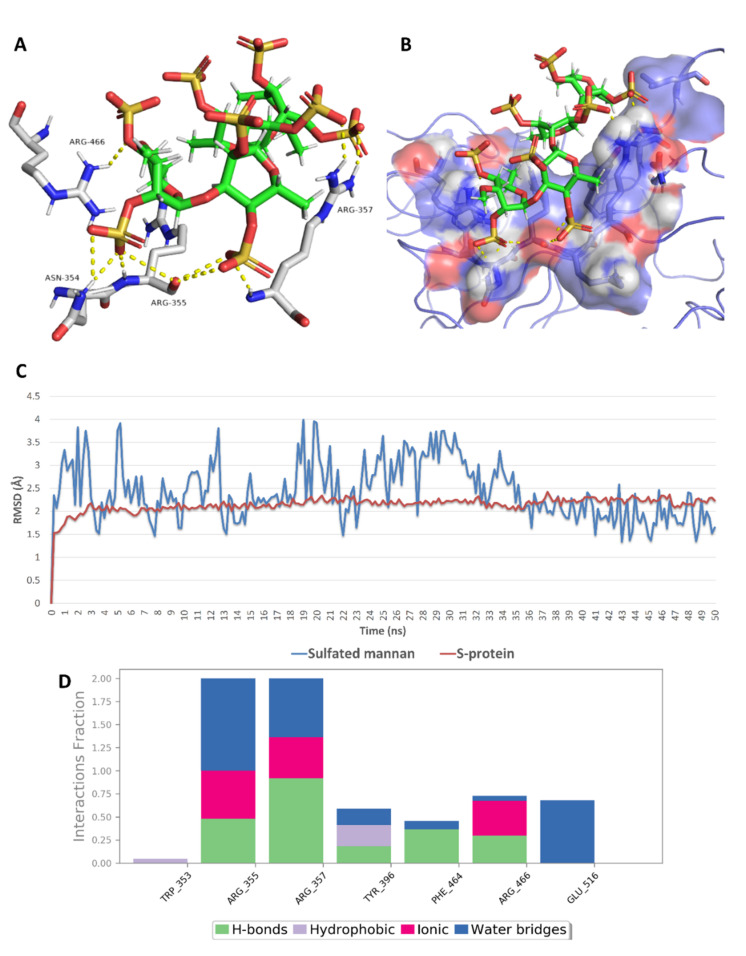
Binding mode of sulfated mannan (compound 3) inside site 2 (**A**,**B**) together with its RMSDs and protein–ligand contacts during 50 ns of MDSs (**C**,**D**).

Regarding site 2, only five sulfated polysaccharides presented docking scores <−5 kcal/mol, of which iota-carrageenan (**4**) was unstable on MDS (got an average RMSD >15 Å) and presented a ΔG value higher than −5 kcal/mol (i.e., 1.7 kcal/mol). Sulfated galactofucan (**1**), sulfated polymannuroguluronate (SPMG) (**2**), and sulfated mannan (**3**) were among the best MSPs that achieved stable binding with S-RBD site 2 and were not previously reported against inhibitory effects of SARS CoV-2 (Table 3). In this site (i.e., site 2), ionic interactions between the negatively charged sulfate moieties and the positively charged key amino acid residues of this site were crucial, along with the other polar interactions (e.g., H-bonding and water bridging), in maintaining stable binding with such shallow binding site (Figure 15, Figure 16 and Figure 17).

**Figure 18:**
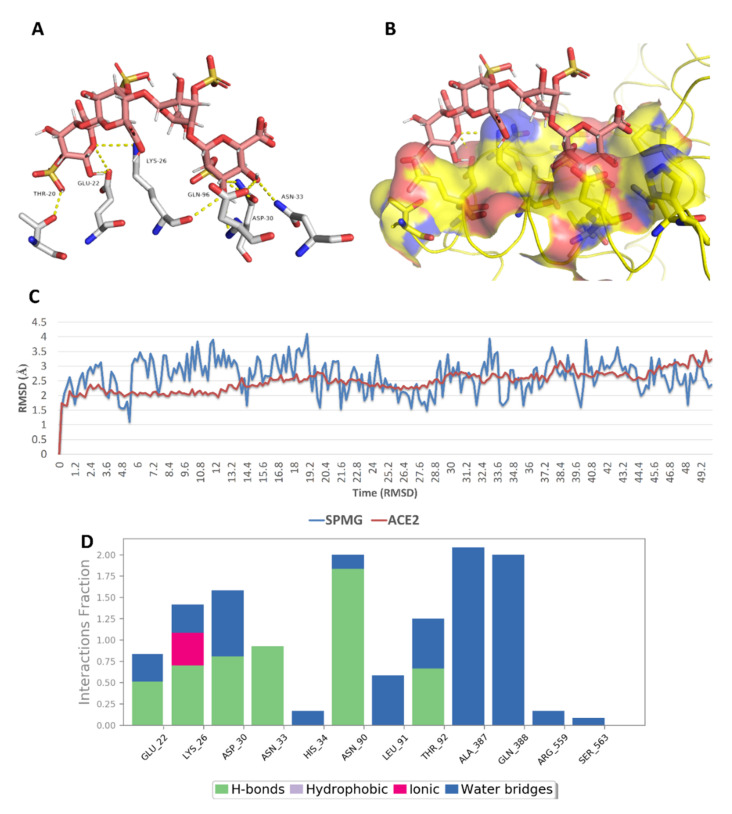
Binding mode of sulfated polymannuroguluronate (SPMG) (compound 2) inside site 3 (**A**,**B**) together with its RMSDs and protein–ligand contacts during 50 ns of MDSs (**C**,**D**).

**Figure 19:**
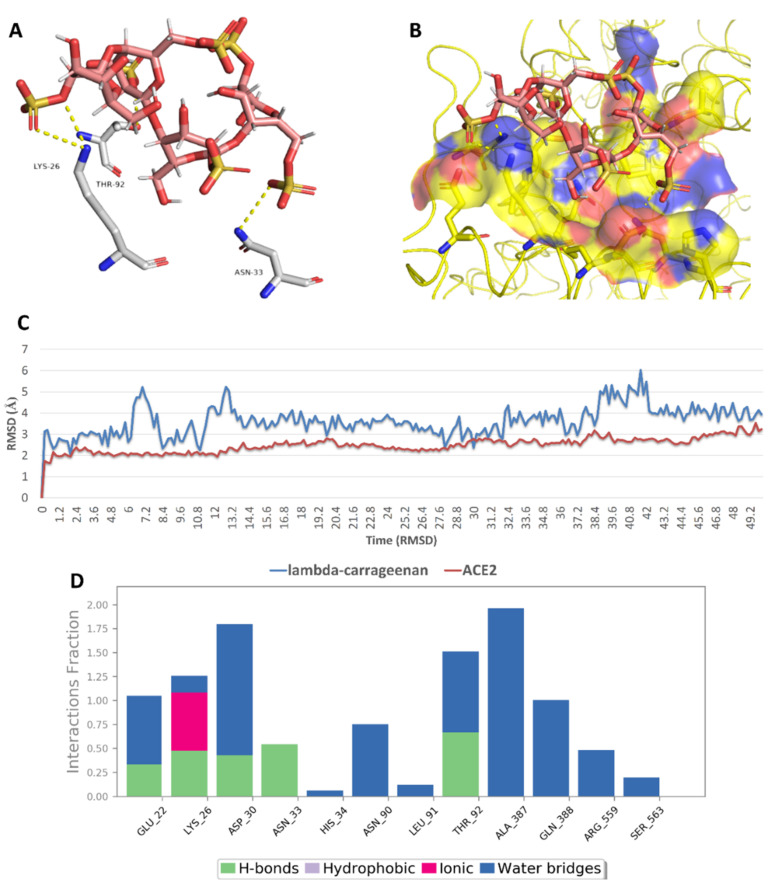
Binding mode of lambda-carrageenan (compound 5) inside site 3 (**A**,**B**) together with its RMSDs and protein–ligand contacts during 50 ns of MDSs (**C**,**D**).

On the other hand, docking against the ACE2 binding site (site 3) revealed that both (SPMG) and lambda-carrageenan were the best-performing MSPs. Additionally, they were also stable over the course of MDS, achieving low deviations from their starting binding poses (RMSD ~ 2.9 Å) and ΔG values <−5 kcal/mol (~−5.2 kcal/mol). Lambda-carrageenan was previously identified to bind to SARS CoV-2 S-protein [44]; however, there have been no reports on the ACE2 binding potential of MSPs so far. As such, focusing on this human protein target will be of great interest in future investigations of SPs as anti-COVID-19 therapeutics. Similarly to site 1 and 2, the dominant interactions between these MSPs and the key amino acid residues inside site 3 were also of the polar type (e.g., H-bonds, water bridges, and ionic interactions) (Figure 18 and Figure 19).

### 2.6. Structure–Activity Relationship

The structural complexity of SPs has made the study of their structure–activity relationship quite challenging, and until now it has not been entirely understood; however, certain key bioactivity-related structural features could be concluded from the previously reported studies and the present modeling study.

The first structural determinant that may affect the antiviral potential of this class of compounds is the number of negatively charged groups (e.g., sulfates or carboxylates). Polysaccharides with higher numbers of sulfate or carboxylate groups per monosaccharide were more bioactive. Highly sulfated glucans (either natural or chemically synthesized derivatives) were found to be far more bioactive as antiviral agents than those with lower degrees of sulfation, while non-sulfated glucans were practically inactive [107,108]. Moreover, in our modeling study, MSPs with a single sulfate or carboxylate group per monosaccharide did not achieve stable binding with either S-RBD or ACE2 (ΔG values > −5 kcal/mol); hence, we could conclude that electrostatic and other polar interactions mediated by these negatively charged groups play a significant role in stabilizing the binding of this class of compounds to S-RBD and ACE2.

Secondly, the distribution of these negatively charged moieties played an essential role in their antiviral activity. In previous studies, carrageenans with similar degrees of sulfation (50 mol%) but isolated from different sources showed varying antiviral activities [39,109,110]. Different charge densities were proposed to explain these findings, and accordingly to explain the antiviral activity [71]. Our modeling study found that neither iota- nor kappa-carrageenan achieved stable binding with any proposed binding sites. This could be attributed to their relatively low degree of sulfation (one sulfate group per onosaccharide); however, both were reported to be bioactive against SARS CoV-2 and other pathogenic viruses. This could be explained by the unique distribution of these limited sulfate groups, which might cause them to be bioactive via different modes of action. In contrast, sulfated galactan (**7**) has a high degree of sulfation (Figure 8); however, it did not achieve stable binding with the proposed binding sites (ΔG value >−5 kcal/mol). Accordingly, the presence of many negatively charged moieties does not guarantee good antiviral activity without the proper orientations and distribution over the polysaccharide backbone.

Finally, the general chemical structure of the polysaccharides (e.g., the stereochemistry of the glycosidic linkage and that of monosaccharides and the presence of branching points in the main backbone) has a general impact on the degree of their antiviral activity [111].

## 3. Methodology

### 3.1. *Databases* *Used*

The search for MSPs in this study was conducted using SciFinder (https://www.cas.org/solutions/cas-scifinder-discovery-platform/cas-scifinder, accessed on 15 May 2021), Scopus (https://www.scopus.com), Web of Science (https://login.webofknowledge.com), Google Scholar, and DNP (Dictionary of Natural Products) databases. The keywords “antiviral”, “SARS CoV-2”, marine-derived”, and “COVID-19” were paired with “sulphated polysaccharide” to obtain published records up until 2021.

### 3.2. Molecular Modeling

We utilized the carbohydrate modeling protocol used by Sapay and his co-workers in our in silico study of MSPs [112].

#### 3.2.1. Molecular Docking

The docking study was carried out against both the receptor-binding domain (RBD) of SARS CoV-2′s spike protein (S-protein) and human angiotensin-converting enzyme-2 (ACE2) (PDB codes: 6lZG and 1R42, respectively) [113,114]. An Autodock Vina docking machine [105] was used for docking experiments. To determine the best binding site for docking, we docked heparin (i.e., sulphated poly saccharide analogue) against both S-protein and ACE2 using the ClusPro server [14] by uploading each crystal structure to the website and choosing heparin as a ligand. The retrieved docking poses were then arranged from the highest to the lowest scores. We selected the docking pose of the highest score and subjected it to a short molecular dynamic simulation (25 ns) experiment to study the stability of heparin in this pose. Heparin showed acceptable stability in this orientation, where it deviated from the initial pose by an average RMSD of 2.1 Å (Figure 6); hence, the binding site of heparin in this pose was used for docking in the subsequent docking experiments. Heparin docking was carried out using its tetrasaccharide form (i.e., the default form in the ClusPro software). Accordingly, we also used the tetrasaccharide form of each MSP for docking experiments to reduce the computational cost required for docking and molecular dynamic simulations. All results were visualized using PyMol software [115].

#### 3.2.2. Molecular Dynamic Simulation

MD simulation experiments were carried out using the MDS machine in Maestro software, Desmond v. 2. [116,117,118], using its default force field (i.e., OPLS-AA). Protein–ligand systems were constructed using the System Builder function. Thereafter, these systems were embedded in an orthorhombic box consisting of TIP3P water and 0.15 M Na+ and Cl- ions (the default dimensions were used). Subsequently, the prepared systems were energy-minimized and equilibrated for 10 ns. Ligand parameterization was carried out during the system building step according to the OPLS force field. For MD simulations carried out using NAMD software, the parameters and topologies of the ligands were computed using the Charmm36 force field. The online software Ligand Reader and Modeler (http://www.charmm-gui.org/?doc=input/ligandrm, accessed on 15 May 2021) [119] and the VMD plugin Force Field Toolkit (ffTK) [120] were utilized for this regard. The generated parameters and topology files were then loaded into VMD to read the protein–ligand complexes without errors, then the simulation step was performed. MD simulations were run for 50 ns at 310 K in the NPT ensemble with the Nose–Hoover thermostat and Martyna-Tobias-Klein barostat using anisotropic coupling. We selected the best binding poses for each compound as starting co-ordinates to investigate their binding stability and mode of interaction. 

#### 3.2.3. Binding Free Energy Calculations

Binding free energy calculations (∆*G*) were performed using the free energy perturbation (FEP) method [121]. Briefly, this method estimates the binding free energy (i.e., ∆G_binding_) according to the following equation: ∆*G*_binding_ = ∆*G*_Complex_ − ∆*G*_Ligand_. These estimations were derived from separate simulations (NAMD software was used for these experiments). All input files required for simulation by NAMD were papered using the online website Charmm-GUI (https://charmm-gui.org/?doc=input/afes.abinding, accessed on 30 April 2021). Subsequently, we loaded these files into NAMD in order to produce the required simulations. The FEP function in NAMD was used to accomplish this experiment. The equilibration step was achieved in the NPT ensemble at 300 K and 1 atm (1.01325 bar) with Langevin piston pressure (for ”complex” and ”ligand”) in the presence of the TIP3P water model. Then, 10 ns FEP simulations were carried out for each ligand and the last 5 ns of the free energy values was measured for the final free energy estimation [121]. All resulting trajectories were visualized and analyzed by VMD software.

## 4. Conclusions

To challenge highly virulent SARS CoV-2 and its emerging mutations, which have cost millions of lives, there is an urgent need to ramp up drug discovery pipelines within a short timeframe. In order to shorten the drug development cycle, screening of existing chemical libraries holds greater potential than starting from scratch. The levels of structural and functional diversity of MSPs have been widely discussed for their antiviral properties; however, the depth of these activities in terms of possible mechanisms against potential drug targets has been poorly studied. A large number of members belong to the class of SPs being overlooked as potential candidates to suppress the virulence of SARS CoV-2.

In the present investigation, we aimed to point out the most probable binding sites on S-protein’s RBD and ACE2 using a well-known sulfated polysaccharide (i.e., heparin) as a molecular probe. Interestingly, the binding of heparin to these sites led to dissociation of the RBD–ACE2 complex. These MDS-derived results indicated and validated these proposed binding sites as potential targets for further ligands from the same chemical class. Accordingly, these identified binding sites were used in a series of docking experiments using most of the collected MSPs in this report. A number of MSPs were then found to be potential binders to these sites. Most of the identified hits have been reported to exert antiviral activity against SARS CoV-2; however, some of them have not, meaning they are considered very promising candidates for experimental testing. Overall, the present investigation shed light on the huge potential of MSPs as antiviral chemical entities and provided the scientific community hints about their potential against SARS CoV-2, with some important structural information to be utilized in further experimental research.

## Figures and Tables

**Table 1:** The antiviral activity levels and modes of action of different MSPs derived from various marine sources.

Sources	Compounds	Activity	Efficacy	Mode of Action	References
Brown Algae					
*Undaria pinnatifid*	Sulfated galactofucan	HSV-1, HSV-2, and HCMV	IC_50_ = 0.2–1.1 μg/mL	Inhibit virus entry and host cell binding	[14,15]
*Cystoseira indica*	Sulfated Fucans	HSV-1 and HSV-2	IC_50_ = 0.50–2.8 μg/mL	Inhibit adsorption	[16]
*Saccharina japonica*	Fucoidan: RPI-27 and RPI-28 in a complex	SARS-CoV-2	EC_50_ = 8.3 ± 4.6 μg/mL	S-protein binding	[17]
*Padina boryana*	Fucoidan: α-(1→3)-linked	SARS-CoV-2	IC_50_ = 15.6 μg/mL	S-protein binding	[18,19]
*Laminaria japonica*	Sulfated polymannuroguluronate (SPMG)	HIV-1	IC_50_ = 30 μg/mL	Inhibit the virus entry and replication	[20]
*Laminaria japonica*	Sulfated polymannuroguluronate (SPMG)	HIV-1	100 μg/mL	Inhibit HIV-1 entry by suppressing rgp120 binding to sCD4	[21]
*Laminaria japonica*	Sulfated polymannuronate (SPMG)-derived oligosaccharides	HIV-1	ID_50_ = 5.3 nM	Inhibit HIV-1 entry by suppressing rgp120 binding to sCD4	[22]
*Adenocystis utricularis*	Sulfated galactofucan	HSV-1 and HSV-2	IC_50_ = 1.25–2.16 μg/mL	Unknown	[23,24]
*Stoechospermum marginatum*	Sulfated fucan: (1→4)- and (1→3)-linked-α-L-fucopyranosyl residues	HSV-1 and HSV-2	EC_50_ = 0.63–10.0 μg/mL	Inhibit binding of the virus to the host cell receptor	[25,26]
*Sargassum horneri*	Fucoidan	HSV-1, HIV-1, and HCMV	IC_50_ = 1–3.3 μg/mL	Unknown	[27]
*Kjellmaniella crassifolia*	Fucoidan KW	Inf A virus (H1N1, H3N2)	IC_50_ < 6.5 μg/mL	Suppress the replication	[28]
*Sargassum patens*	Galactofucan	HSV-1 and HSV-2	EC_50_ = 1.3–5.5 μg/mL	Inhibit adsorption	[29]
*Leathesia difformis*	Fucoidan	HSV-1, HSV-2, and HCMV	IC_50_ = 0.5–1.9 μg/mL	Inhibit protein synthesis and adsorption	[30]
*Fucus evanescens*	Fucoidan	HSV-2	10 mg/kg/day for 5 days	Unknown	[31]
*Undaria pinnatifida*	Fucoidan	HSV-1, HSV-2, and HCMV	IC_50_ = 1.5–2.6 μg/mL	Inhibit entry and binding	[32]
*Sphacelaria indica*	Xylogalactofucan and alginic acid	HSV-1	IC_50_ = 0.6–10 μg/mL	Inhibit adsorption	[33]
*Sargassum mcclurei, Sargassum polycystum,* and *Turbinara ornata*	Fucoidan	HIV-1	IC_50_ = 0.33–0.7 μg/mL	Inhibit entry	[34]
*Scinaia hatei*	Sulfated xylomannan	HSV-1 and HSV-2	IC_50_ = 0.5–4.6 μg/mL	Inhibit replication and virus binding	[16]
*Cladosiphon okamuranus*	Fucoidan	NDV	IC_50_ = 0.75 ± 1.6 μg/mL	Inhibit viral-induced syncytia formation	[35]
*Cladosiphon okamuranus*	Fucoidan	DENV-2	Structure-based analysis: fucoidan interacts directly with envelope glycoprotein (EGP) on DEN2	Inhibit binding	[36]
*Undaria pinnatifida-* derived fucoidan (UPF)	Fucoidan	InfAV (H1N1, PR8)	7.0 mg/day for 7 days		[37]
*Undaria pinnatifida*	Fucoidan	Avian InfAV	5 mg/day for 14 days	Decrease replication	[38]
Red Algae					
*Gymnogongrus griffithsiae* and *Cryptonemia crenulata*	Kappa/iota/nu Carrageenan and DL-galactan hybrid	HSV-1 and HSV-2	IC_50_ = 0.5–5.6 μg/mL	Inhibit adsorption	[39]
*Sebdenia polydactyla*	Sulfated xylomannans	HSV-1	IC_50_ = 0.35–2.8 μg/mL	Inhibit virus attachment to the host cell	[40]
*Euchema spinosum*	Iota-carrageenan	SARS-CoV-2	IC_50_ = 2.6 μg/mL	Inhibit replication	[41,42]
*Euchema spinosum*	Iota-carrageenan with Xylitol^®^ nasal spray	SARS-CoV-2	IC_50_ < 6.0 μg/mL	Inhibit SARS-CoV-2 in vitro	[42,43]
*Chondrus crispus*	lambda-carrageenan	SARS-CoV-2, and InfV A and B	EC_50_ = 0.3–1.4 μg/mL	Prevent the entry	[42,44]
*Euchema spinosum*	Iota-carrageenan containing Xylometazoline HCL	hRV1a, hRV8, and hCoV-OC43	IC_50_ for hRV1a and hRV8 = 1.56–6.2 μg/mLMIC for hCoV-OC43 = 0.024 μg/mL	Prevent the entry	[7,42]
*Gigartina skottsbergii*	κ/ι /μ/ν-carrageenan	HIV-1	IC_50_ = 0.4–3.3 μg/m	Prevent binding and replication of virions	[45]
*Gymnogongrus torulosus*	DL-hybrid sulfated galactan	HSV-1 and HSV-2	IC_50_ = 1.1 to 27.4 μg/mL	Unknown	[46,47]
*Cryptonemia crenulata*	DL-galactan hybrid C2S-3	DENV-2	IC_50_ = 1 μg/mL	Block virus multiplication	[48,49]
*Stenogramme interrupta*	ξ- and λ-carrageenans	HSV-1 and HSV-2	IC_50_ = 0.65–2.88 μg/mL	Inhibit binding and replication	[50]
*Gracilaria corticata*	Sulfated galactan	HSV-1 and HSV-2	IC_50_ = 0.19–0.24 μg/mL	Inhibit virus attachment to the host cell	[51,52]
*Aghardhiella tenera*	Sulfated galactan	HIV-1 and HIV-2	IC_50_ = 0.05–0.5 μg/mL	Inhibit cytopathic effect of HIV-1 and HIV-2 in MT-4 cells	[53]
*Nemalion helminthoides*	Sulfated mannan	HSV-1	IC_50_ = 5.43–9.68 μg/mL	Unknown	[54]
*Nothogenia fastigiata*	Sulfated xylomannan	HSV-1 and HSV-2	IC_50_ = 0.6–1.3 μg/mL	Prevent binding and replication	[55,56]
*Scinaia hatei*	Sulfated xylomannan	HSV	IC_50_ = 0.5 μg/mL	Inhibit replication	[57]
*Nothogenia fastigiata*	Sulfated xylogalactans	HSV-1	EC_50_ = 15.0–32.6 μg/mL	Inhibit virus attachment to the host cell	[58,59]
*Euchema spinosum* *Chondrus crispus*	Lambda/Iota-carrageenan	DENV-2 and DENV-3	EC_50_ = 0.14–4.1 μg/mL	Interference with virus adsorption	[42,60]
*Euchema spinosum*	Iota-carrageenan nasal spray	hRV, hCV, and InfA	3 times per day for 7 days	Inhibit virus attachment to the host cell	[6,42]
*Euchema spinosum*	Iota-carrageenan containing lozenges (Coldamaris^®^)	hRV1a, CoV OC43, and InfA H1N1n	10 mg daily	Prevent binding and inhibit replication	[8]
*Euchema spinosum*	Iota-carrageenan nasal spray	hRV1a, CoV OC43, and InfA H1N1n	Total of 1.0 mg daily	Prevent the virus from binding to cell surfaces or penetrating the cells	[42,61]
*Euchema spinosum*	Iota-carrageenan, nasal spray	H1N1 and H3N2	IC_50_ = 0.04–0.2 μg/mL	Effectively inhibit virus adsorption to host cells and reduce replication	[42,62]
*Euchema spinosum*	Iota-Carrageenan	hRV	10^4^–10^7^ TCID_50_/mL; 5–50 μg/mL	Prevent binding and/or the entry into the cells, inhibit replication	[42,63]
*Euchema spinosum*	Iota-Carrageenan	HPV	IC_50_ = 0.005 μg/mL	Blocking the initial interaction of capsids with cells and exert a postattachment inhibitory effect.	[64]
*Acanthophora specifira*	Lambda-carrageenan	HSV-1 and RVFV	IC_50_ = 75.8–80.5 μg/mL	Inhibit replication	[65]
*Lithothamnion muelleri*	Sulfated xylogalactans	HSV-1 and HSV-2	EC_50_ = 49.64–125.79 μg/mL	Inhibit adsorption and penetration	[59,66]
*Scinaia hatei*	Sulfated xylan	HSV-1 and HSV-2	IC_50_ = 0.22–1.37 μg/mL	Inhibit entry	[67]
*Euchema spinosum*	Iota-Carrageenan	SARS-COV-2	IC_50_ ≥ 125 μg/mL	Unknown	[18,42]
Sigma (Sigma Aldrich)	Carrageenan	Japanese encephalitis virus (JEV)	EC_50_ = 15 µg/mL	Inhibit attachment and the cellular entry stages	[68]
*Tichocarpus crinitus*	κ/β-carrageenan	Tobacco Mosaic Virus (TMV)	1 mg/mL	Significantly inhibit virus replication	[69]
*Chondrus crispus*	Lambda-carrageenan	DENV-2 and DENV-3	>99% reduction in virus production at 20 μg/mL	Inhibit entry	[42,70]
*Meristiella gelidium*	Iota/kappa/nu-hybrid carrageenan	DENV-2	IC_50_ = 0.14–1.6 μg/mL	Unknown	[71]
	Iota/lambda/kappa-carrageenan	Hepatitis A Virus (HAV)	Ratio of CD_50_ to ED_50_ >400	Inhibit replication	[72]
*Sphaerococcus coronopifolius* and *Boergeseniella thuyoides*	Sulfated galactan	HIV-1 and HSV-1	EC_50_ = 4.1–17.2 μg/mL	Inhibit adsorption and replication	[73]
Green Algae					
*Enteromorpha compressa*	Ulvan	HSV	IC_50_ = 28.25 μg/mL	Inhibit adsorption and replication	[74,75]
*Monostroma latissimum*	Sulfated rhamnan	EV71	IC_50_ = 0.5 μg/mL	inhibit replication	[76]
*Ulva intestinalis*	Ulvan	Measles virus (MeV)	IC_50_ = 3.6 μg/mL	Reduce the formation of syncytia	[77]
*Ulva clathrata*	Ulvan	NDV	IC_50_ = 0.1 μg/mL	Inhibit cell-cell fusion via a direct effect on F0 protein	[78]
*Ulva armoricana*	Ulvan (enzymatic preparations)	HSV-1	EC_50_ = 320.9–373.0 μg/mL	Unknown	[79]
*Monostroma nitidum*	Rhamnan Sulfate	HSV-1, HSV-2, HCMV, MeV, MuV, HCV, HCoV and HIV	EC_50_ = 0.77–8.30 μg/mL	Inhibit viral proliferation	[80]
*Monostroma nitidum*	Rhamnan Sulfate	HSV-2	IC_50_ = 0.87 μg/mL	Suggested to inhibit adsorption or penetration	[81]
*Gayralia oxysperma*	Sulfated heterorhamnan	HSV-1 and HSV-2	IC_50_ = 0.036–0.3 μg/mL	Inhibit multiplication	[82]
*Codium fragile*	Sulfated galactan	HSV-2	IC_50_ = 4.7 μg/mL	Interference with the early steps such as virus adsorption to and penetration into host cells	[83]
*Monostroma nitidum, C. okamurai, C. scapelliformis, Chaetomorpha crassa, C. spiralis, Codium adhaerens, C. fragile, Caulerpa brachypus* and *Caulerpa latum*	Sulfated arabinoxylogalactan	HSV-1	IC_50_ = 0.38–8.5 μg/mL	Inhibit binding, penetration, and the late stages of replication	[84]
*Enteromorpha compressa*	Sulfated heteroglycuronan (chemically modified)	HSV-1	IC_50_ = 28.25 μg/mL	Inhibit replication	[74]
*Ulva lactuca*	Ulva sulfated polysaccharide extracts	JEV	0.75 mg/day for 7 days	The survival rate significantly increased	[85]
*Ulva Pertusa*	Ulvan	Avian InfAV	50 mg twice (INJ) immunizations dose	Enhance AIV-specific antibody production and improve the humoral immunity level	[86]
*Caulerpa racemosa*	Sulfated heteropolysaccharide	DENV-2	IC_50_ = 0.6 μg/mL	Interfere with virus multiplication	[87]
Blue-Green Algae					
*Spirulina platensis*	Calcium Spirulan (Ca-SP)	HSV-1, HCMV, MeV, MuV, InfA, HIV-1	EC_50_ = 0.92–23 μg/mL	Inhibit replication and penetration	[88]
*Spirulina platensis*	Calcium spirulan, Sodium spirulan, and potassium spirulan	HSV-1	IC_50_ = 0.46–0.88 μg/mL	Inhibit replication	[89]
*Spirulina platensis*	Spirularin^®^ HS “Ocean Pharma (Topical cream containing Ca-SP in combination with SPME)	HSV-1	15 mg/g twice daily for 3 weeks(10 mg/g SPME)	Significantly prevent herpes labialis exacerbation	[90]
*Aphanothece sacrum*	Sulfated polysaccharides (ASWPH)	HSV-2 and Inf A (H1N1)	IC_50_ = 0.32–1.2 μg/mL	Inhibit adsorption	[91]
*Spirulina maxima*	Hot water extract (HWE)	HSV-1, HSV-2, HCMV, and pseudorabies virus (PRV)	ED_50_ = 0.069–0.333 mg/mL	Inhibit adsorption and penetration	[92]
Microalgae					
Red; *Porphyridium sp.*	cell-wall sulphated polysaccharide	HSV-1, HSV-2 and Varicella Zoster Virus (VZV)	IC_50_ = 1 µg/mL	Prevent adsorption and/or inhibit the production of new viral particles	[93]
Diatom; *Navicula directa*	Naviculan	HSV-1, HSV-2, HIV, and Inf A	IC_50_ = 7.4–170 µg/mL	Inhibit binding and penetration. Inhibit cell–cell fusion between HIV gp160- and CD4.	[94]
*Gyrodinium impudium*	P-KG03	InfA H1N1 and H3N2	EC_50_ = 0.19–0.48 μg/mL	Inhibit replication and entry	[95]
*Gyrodinium impudicum*	P-KG03	EMCV	EC_50_ = 26.9 µg/ml	Inhibit replication	[96]
*Cochlodinium polykrikoides*	Extracellular sulfated polysaccharides (A1 and A2)	HIV-1, HSV-1, Inf A, RSV-A, and RSV-B	EC_50_ = 1.1–4.52 µg/mL	Inhibit replication	[97]
Marine animal-derived sulfated polysaccharides					
*Thelenota ananas*	Sea cucumber Fucosylated chondroitin sulfate	HIV-1	EC_50_ = 0.73–31.86 μg/mL	inhibit the entry and replication	[98]
*Stichopus japonicus*	Sea cucumber sulfated polysaccharide (SCSP)	SARS-COV-2	EC_50_ = 9.10 μg/mL	Interact with the S glycoprotein	[18]
Squid cartilage	Chondroitin sulphate E (CS-E)	DENV	EC_50_ = 0.52–11.8 μg/mL	Inhibit the entry via targeting E protein	[99]
Squid cartilage	Chondroitin sulfate E (CS-E)	HSV-1 and HSV-2	IC_50_ = 0.06 to 0.2 μg/mL	Inhibit binding	[100]

**Table 2:** Docking scores and binding free energies of the top-scoring MSPS against S-RBD of the original and mutated SARS CoV-2 S-protein (site 1).

No.	Compound	Vina Score (kcal/mol) ^#^		Δ*G* *		Average RMSD (Å)		Reported Activity Against SARS CoV-2
		Original Strain	Mutated Strain **	Original Strain	Mutated Strain **	Original Strain	Mutated Strain **	
1	Sulfated galactofucan: α- (1,3)- and (1,4)-α-L- (alternating)	−6.3	−6.4	−6.0	−6.3	5.1	4.5	No
2	Sulfated polymannuroguluronate (SPMG)	−7.5	−6.4	−7.3	−5.9	2.6	4.1	Yes
3	Sulfated mannan	−7.6	−7.7	−7.2	−7.4	2.3	1.9	No
4	iota-carrageenan	−6.0	−6.0	−0.8	−0.9	>15 ***	>15 ***	Yes
5	lambda-carrageenan	−7.0	−7.0	−5.4	−5.8	4.6	4.3	Yes
6	kappa-carrageenan	−6.6	−6.4	−1.3	−1.9	>15 ***	>15 ***	Yes
7	Sulfated galactan	−6.2	−6.3	−0.4	−0.1	>15 ***	>15 ***	No
8	Sulfated heterorhamnan	−6.1	−6.2	−0.1	−0.7	>15 ***	>15 ***	No
9	Chondroitin sulphate E (CS-E)	−7.6	−7.5	−7.1	−6.1	1.1	2.8	No
10	Heparin	−6.5	−6.4	−6.1	−6.1	3.3	3.3	Yes

Note: * Δ*G* was calculated using FEP method (see Materials and Methods for further information). ** N501Y-mutated strain of SARS CoV-2. *** This compound dissociated early at the beginning of MDS experiments (at ~25 ns). # The reported scores are the averages of three independent docking experiments (standard errors were between 0.1 and 0.3).

**Table 3:** Docking scores and binding free energies of top-scoring MSPS against RBD of SARS CoV-2 S-protein (site 2).

No.	Compound	Vina Score (kcal/mol)	Δ*G* *	Average RMSD (Å)	Reported Activity Against SARS CoV-2
1	Sulfated galactofucan: α- (1,3)- and (1,4)-α-L- (alternating)	−5.3	−5.7	2.4	No
2	Sulfated polymannuroguluronate (SPMG)	−5.5	−5.9	2.1	No
3	Sulfated mannan	−5.4	−5.1	2.6	No
4	iota-carrageenan	−5.0	−1.7	>15 **	Yes
5	lambda-carrageenan	−5.5	−5.4	2.5	Yes
10	Heparin	−5.0	−5.8	1.9	Yes

Note: * Δ*G* was calculated using the FEP method (see Materials and Methods for further information). ** This compound dissociated early at the beginning of MDS (at ~25 ns).

**Table 4:** Docking scores and binding free energies of the top-scoring MSPS against ACE2 (site 3).

No.	Compound	Vina Score (kcal/mol)	Δ*G* *	Average RMSD (Å)	Reported Activity Against SARS CoV-2
2	Sulfated polymannuroguluronate (SPMG)	−5.9	−5.4	2.8	No
5	lambda -carrageenan	−5.5	−5.0	3.1	Yes
10	Heparin	−5.3	−5.0	3.0	Yes

Note: * Δ*G* was calculated using FEP method (see Materials and Methods for further information).

## Data Availability

Not applicable.

## Supplementary Materials

The following are available online at https://www.mdpi.com/article/10.3390/md19080406/s1, Figure S1–S8 indicating details about the reported SMPs in addition to the reported SMPs that were reported in the manuscripts.
