# Supplementary material for: Marine Sulfated Polysaccharides as Promising Antiviral Agents: A Comprehensive Report and Modeling Study Focusing on SARS CoV-2
Source: Mar Drugs. 2021 Jul 22;19(8):406. doi: 10.3390/md19080406 (PMC8401819; doi:10.3390/md19080406)
Supplement: Supplementary file 1 [file marinedrugs-19-00406-s001.zip › marinedrugs-1261170-supplementary.pdf]

Supplementary data

Marine Sulfated Polysaccharides as Promising Antiviral Agents: A Comprehensive Review and Modelling Study Focussing on SARS CoV-2

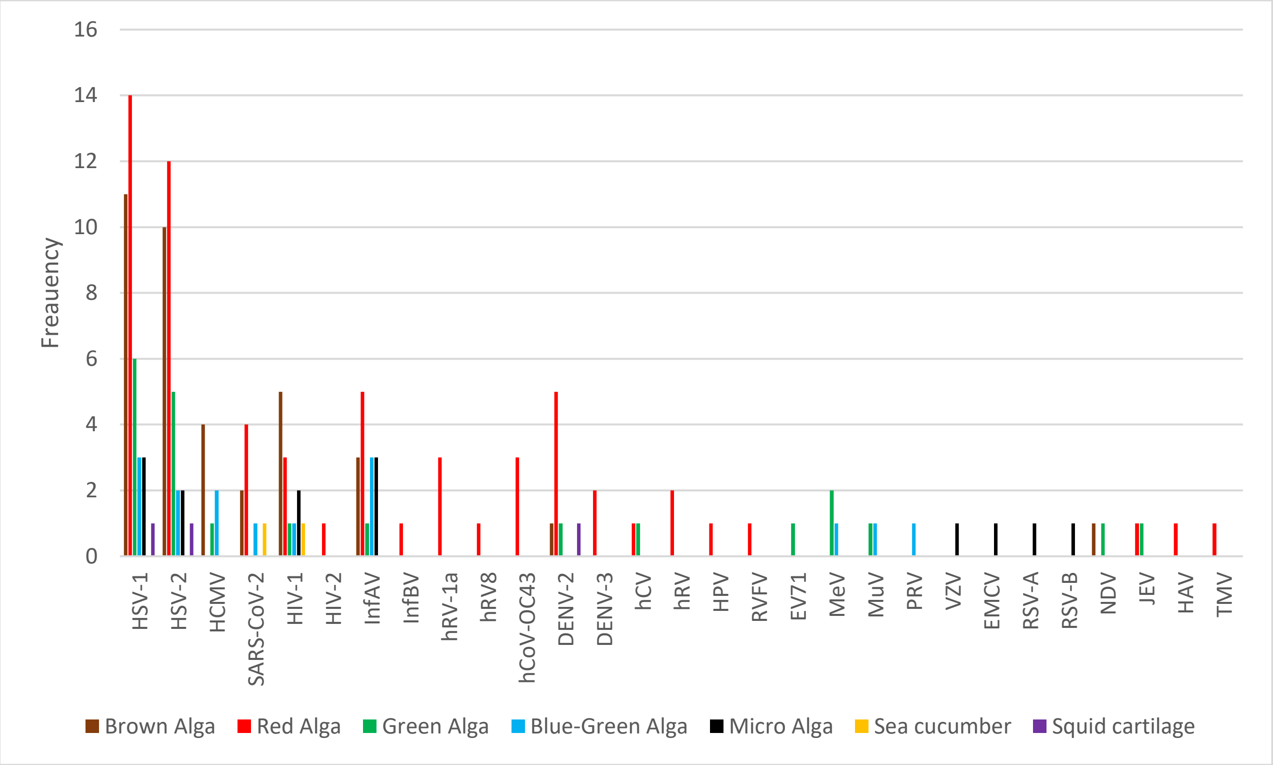

Figure S1. The frequent study of marine sources of MSPs that have shown antiviral activities against the mentioned virus strains during the last 25 years.

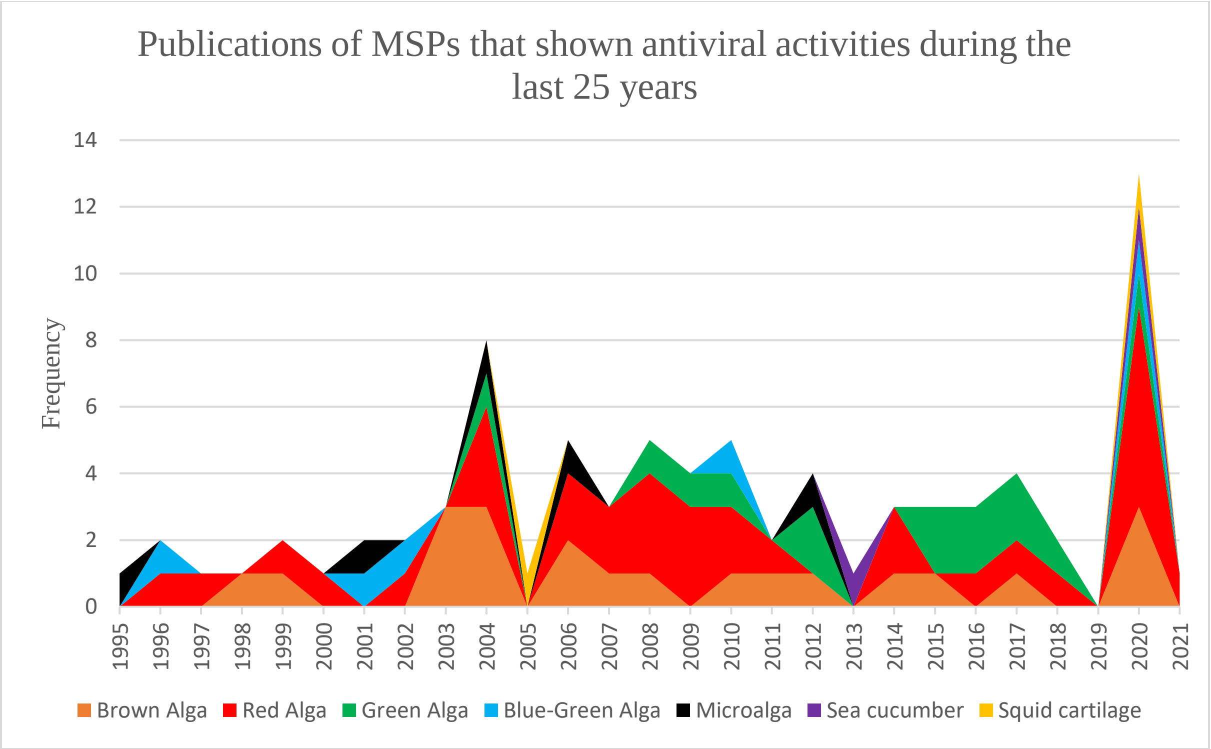

Figure S2. The publications number of MSPs that exhibit antiviral activities during the last 25 years.

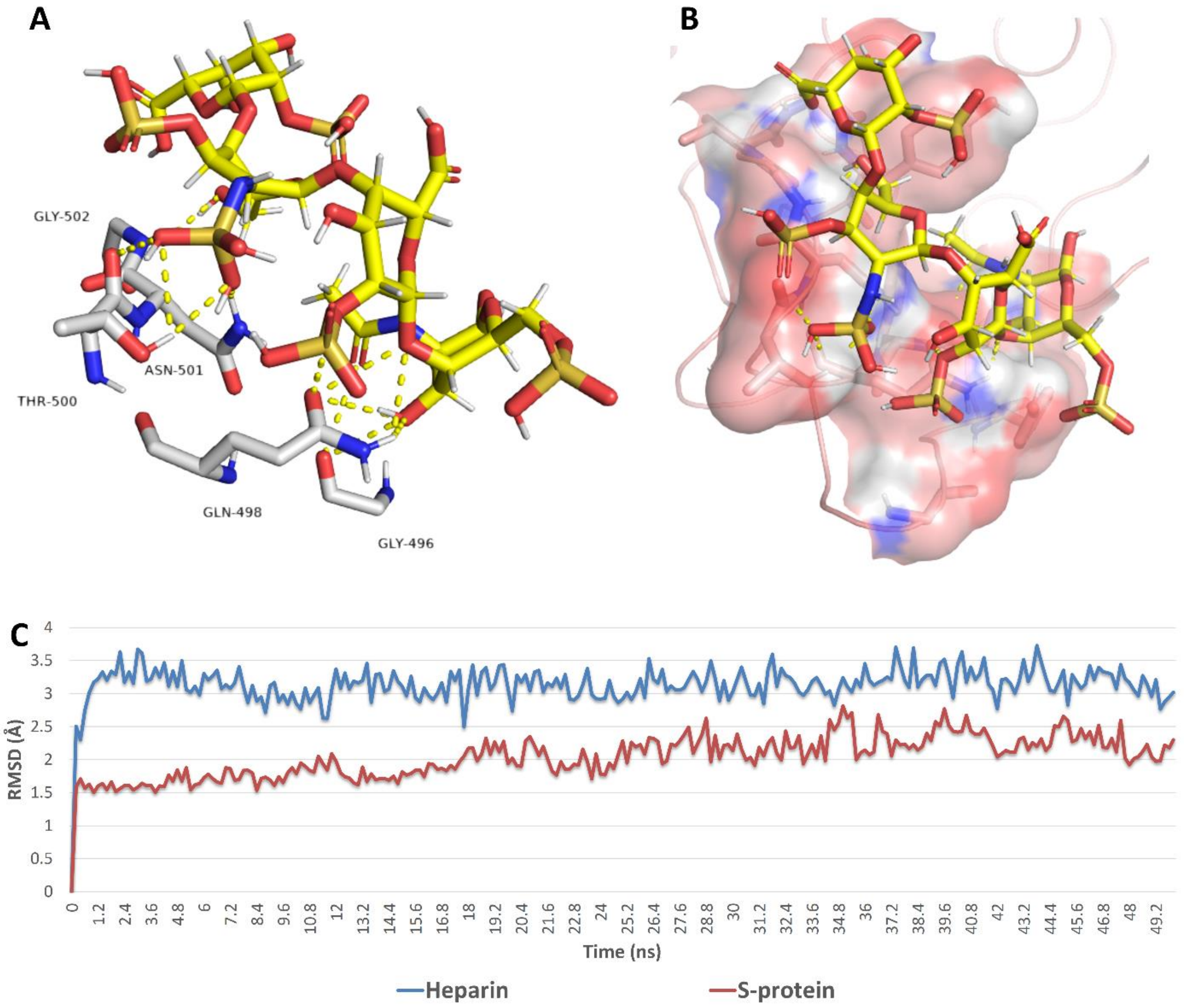

Figure S3. Binding mode of heparin with Site 1 (A and B) together with the RMSDs of heparin and RBD during 50 ns MDSs (C).

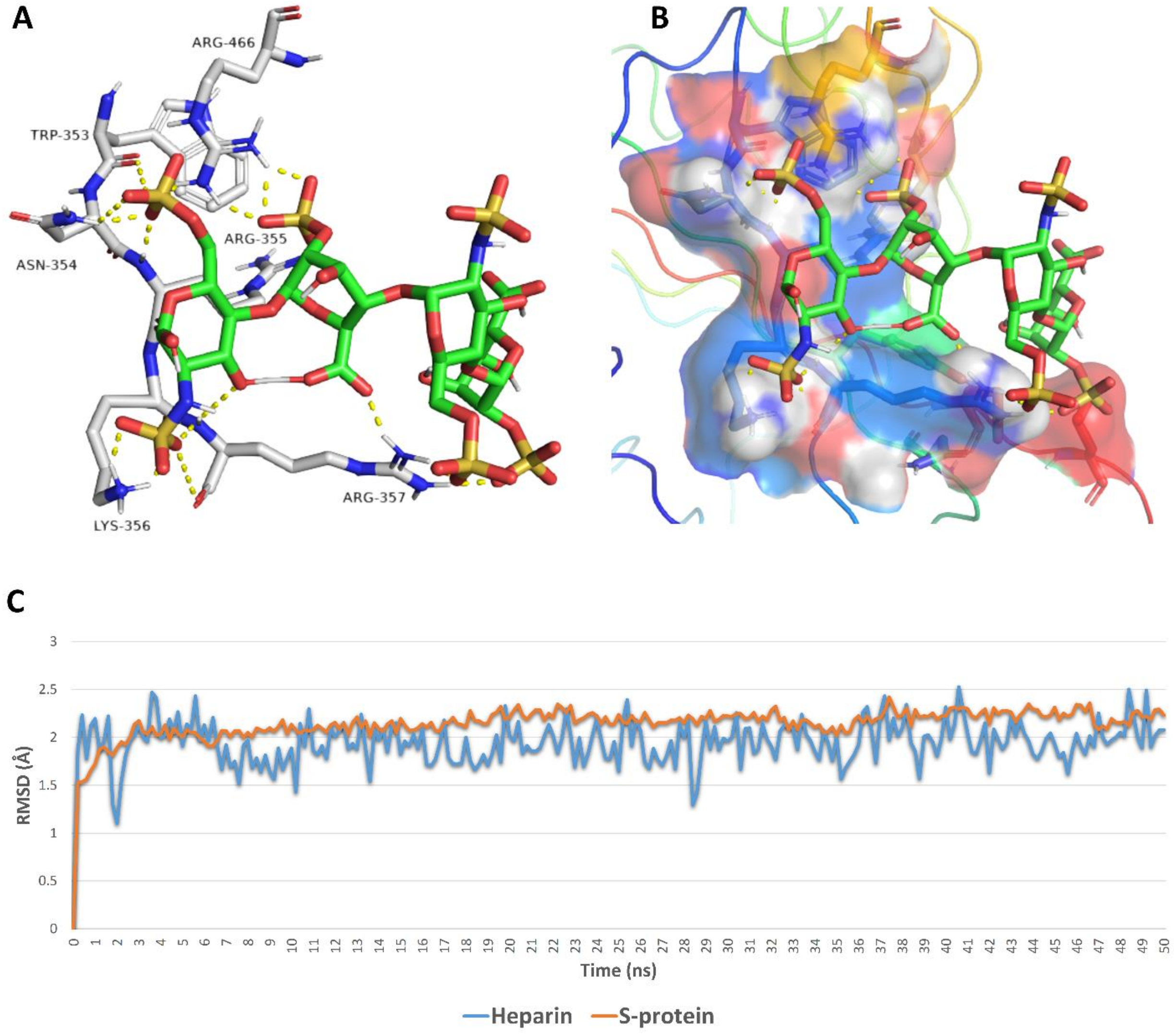

Figure S4. Binding mode of heparin with Site 2 (A and B) together with the RMSDs of heparin and RBD during 50 ns MDSs (C).

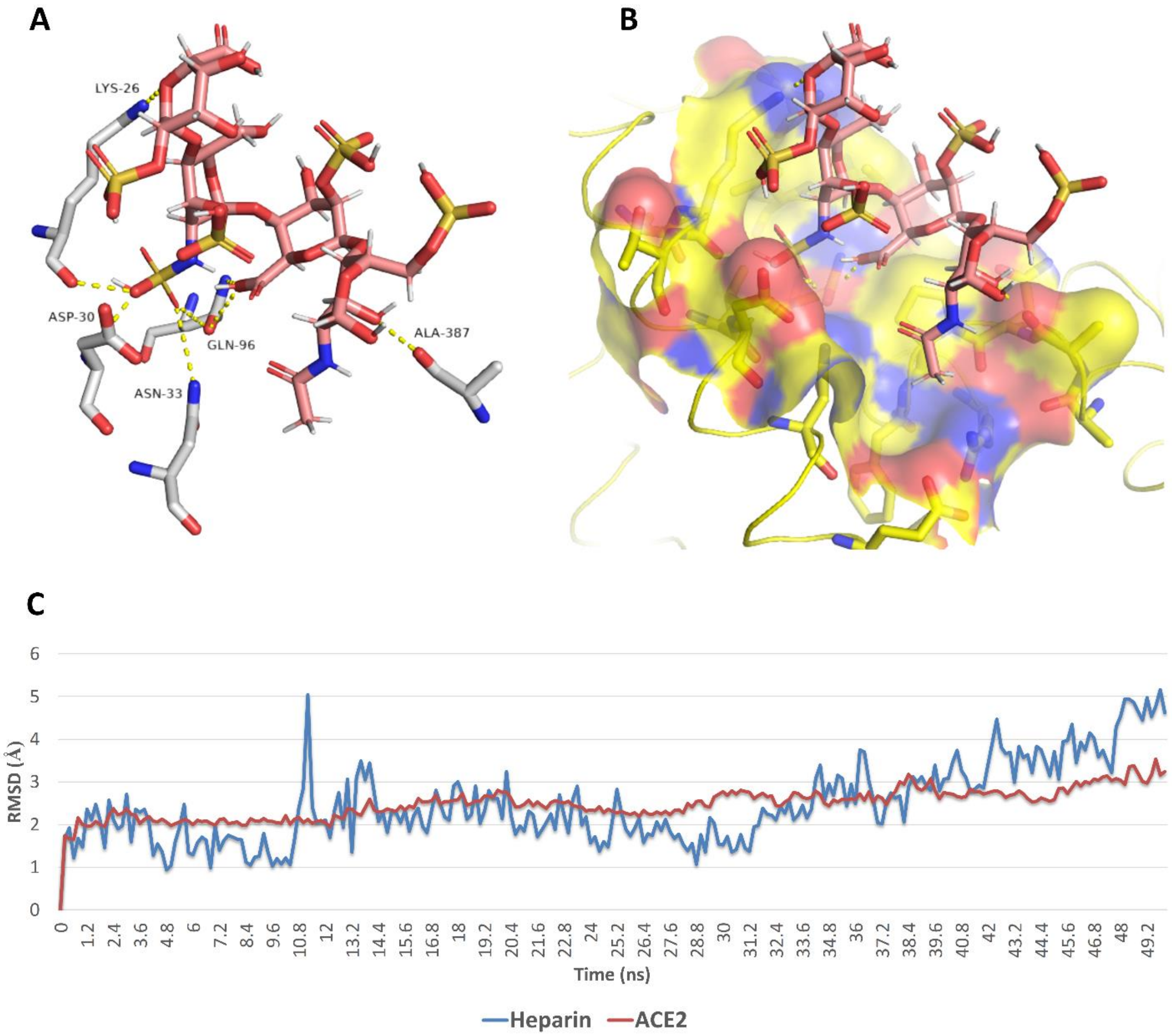

Figure S5. Binding mode of heparin with Site 3 (A and B) together with the RMSDs of heparin and RBD during 50 ns MDSs (C).

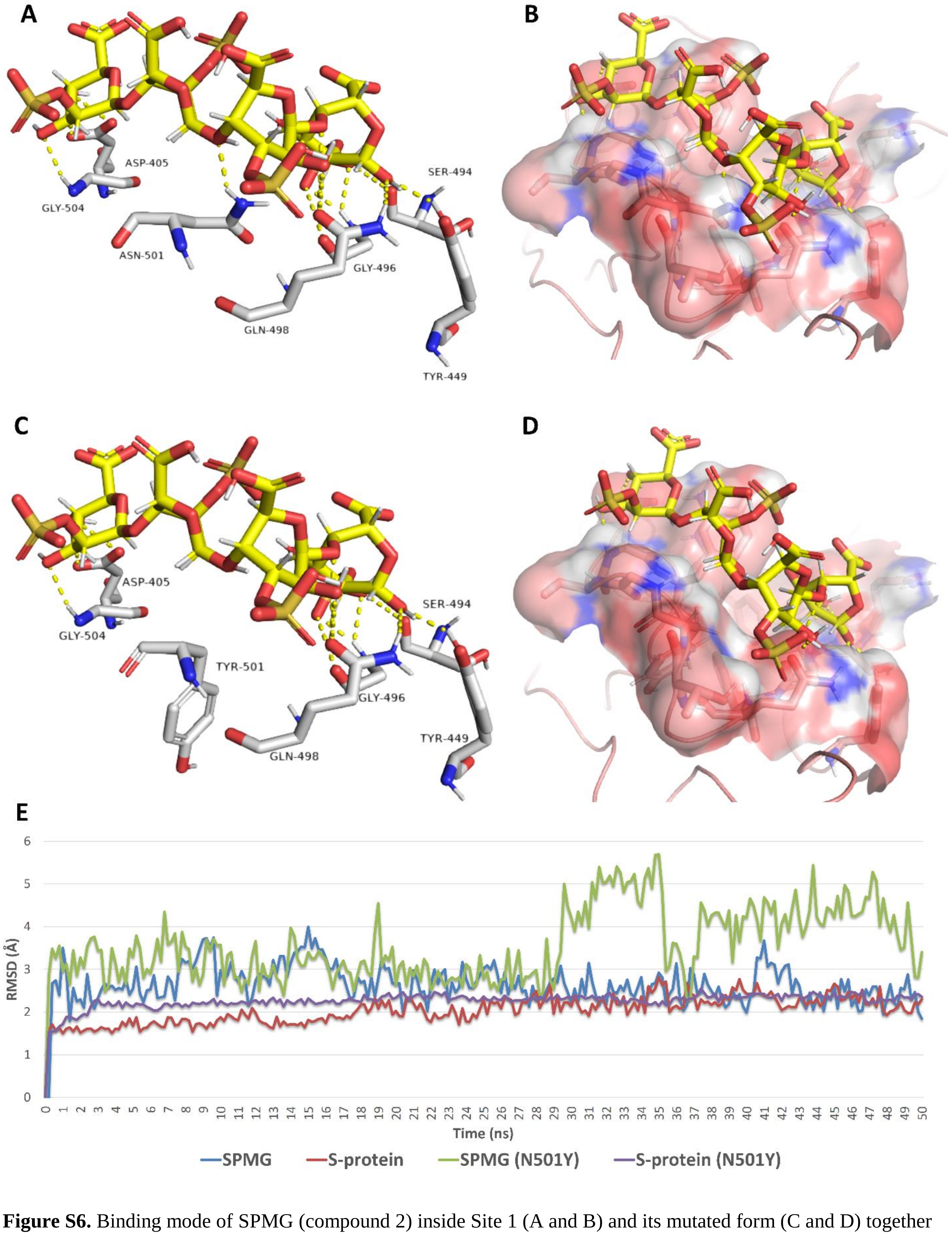

Figure S6. Binding mode of SPMG (compound 2) inside Site 1 (A and B) and its mutated form (C and D) together with its RMSDs during 50 ns of MDSs (E).

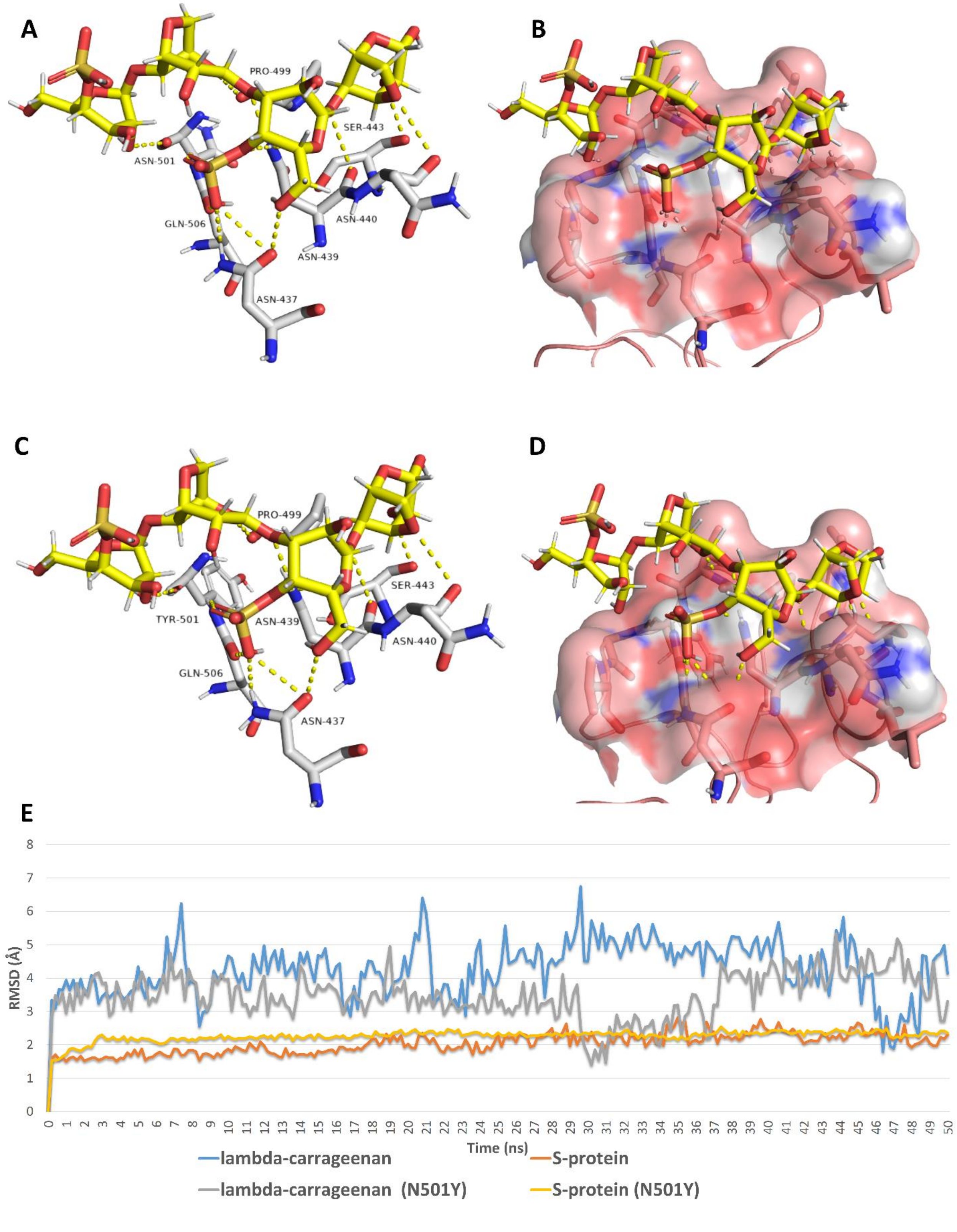

Figure S7. Binding mode of lambda-carrageenan (compound 5) inside Site 1 (A and B) and its mutated form (C and D) together with its RMSDs during 50 ns of MDSs (E).

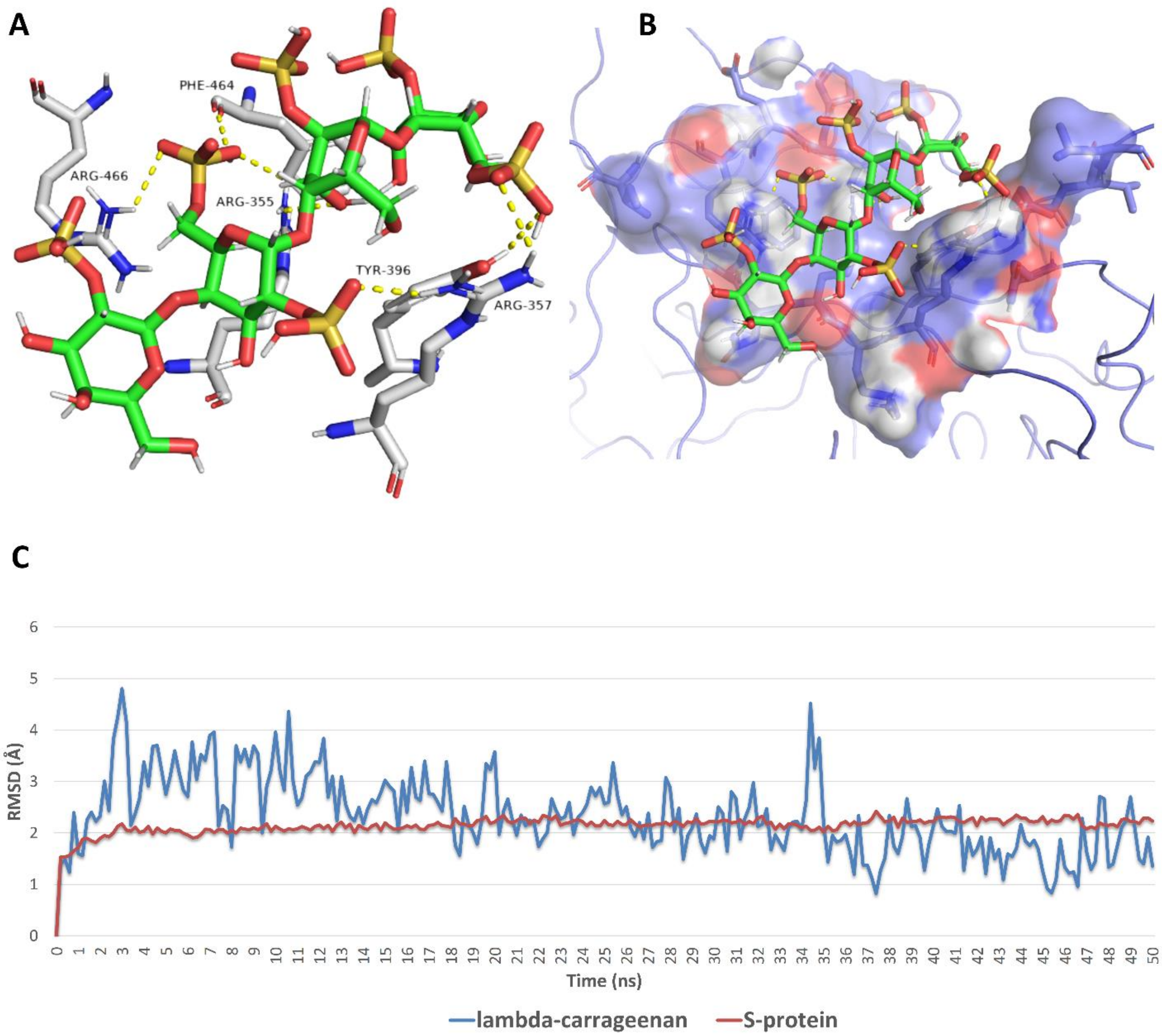

Figure S8. Binding mode of lambda-carrageenan (compound 5) inside Site 2 (A and B) together with its RMSDs during 50 ns of MDSs (C).

Brown Algae

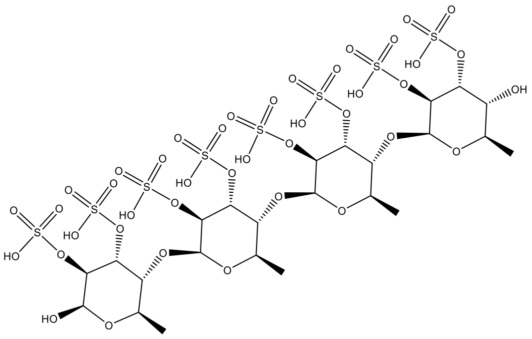

Fucoidan: α-(1 → 4)-linked glucopyranosyl residues

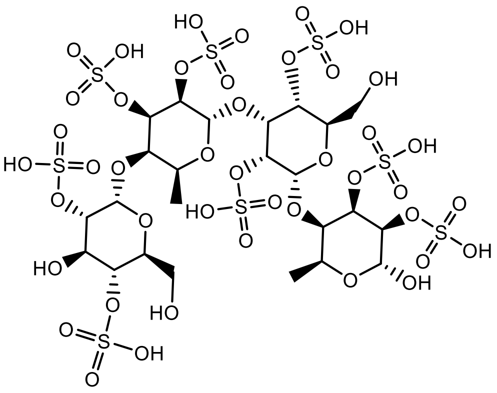

Sulfated galactofucan: α- (1,3)- and (1,4)-α-L- (alternating)

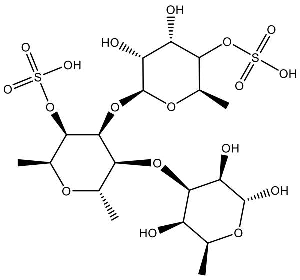

Sulfated Fucans

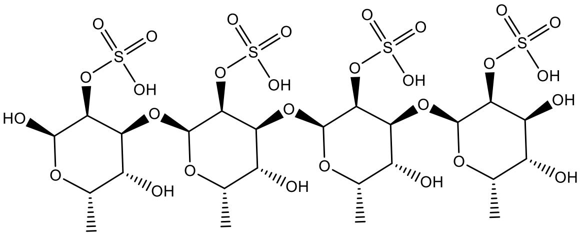

Fucoidan: α-(1→3)-linked

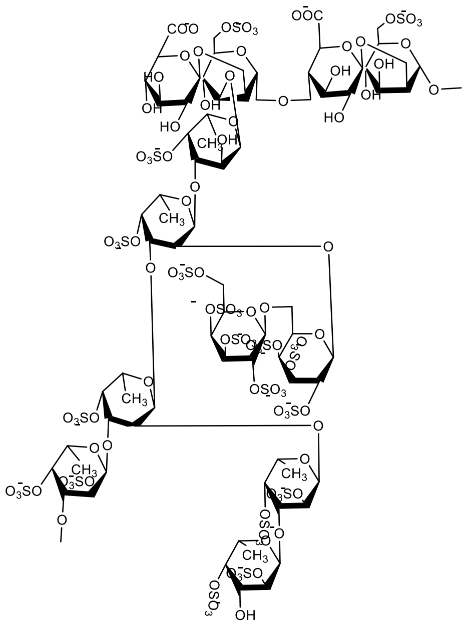

Fucoidan: RPI-27 and RPI-28 in a complex

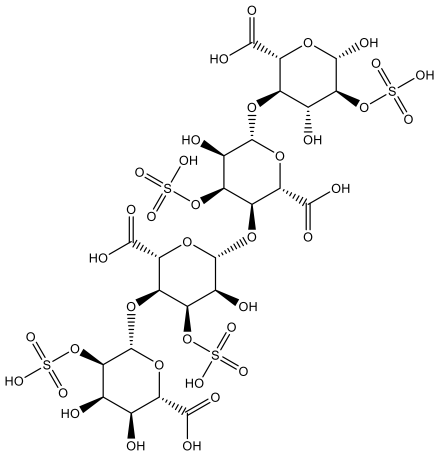

Sulfated polymannuroguluronate (SPMG)

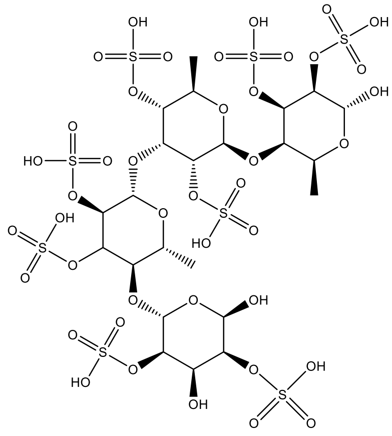

Sulfated galactofucan

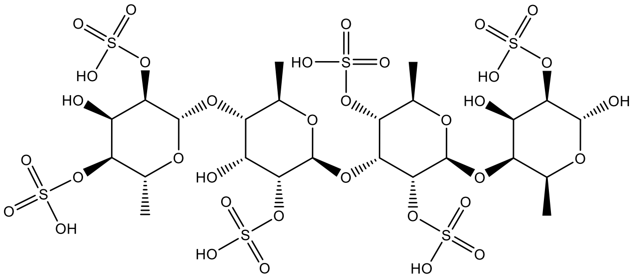

Sulfated fucan: (1→4)- and (1→3)-linked-α-l-fucopyranosyl residues

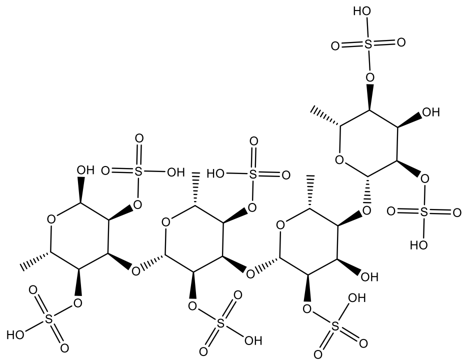

Fucoidan: (1,3)-α-l-fuc, (1,3)- and (1,4)-α-l-fuc

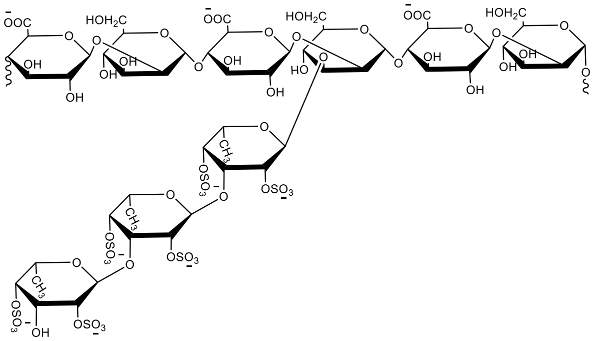

Fucoidan KW

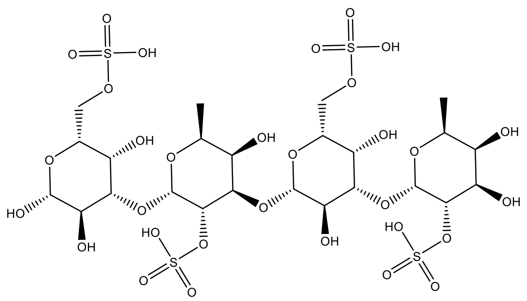

Galactofucan

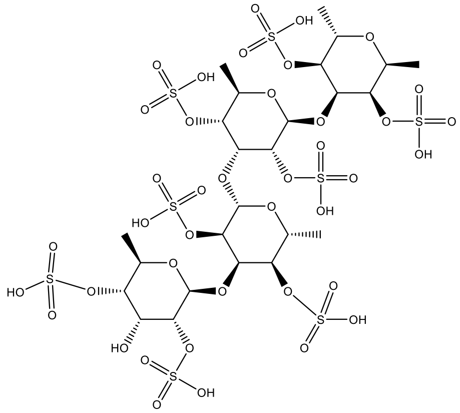

Fucoidan

Red Algae

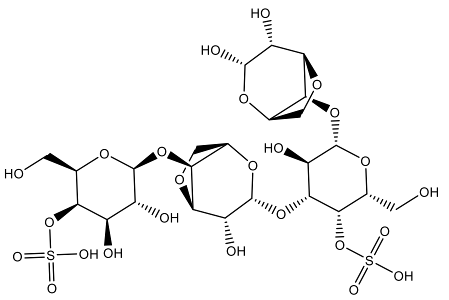

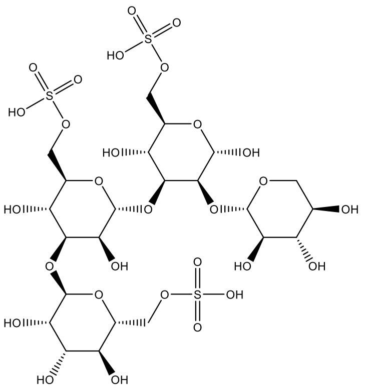

Kappa-Carrageenan

Sulfated xylomannans

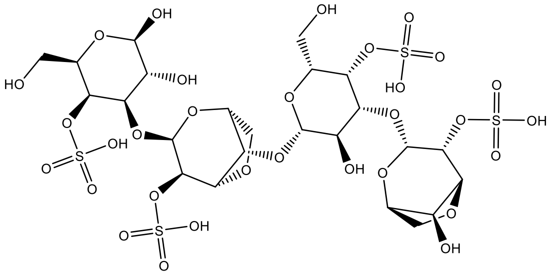

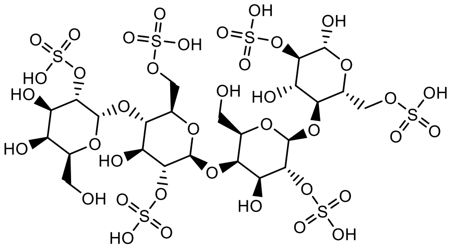

Iota-carrageenan

lambda-carrageenan

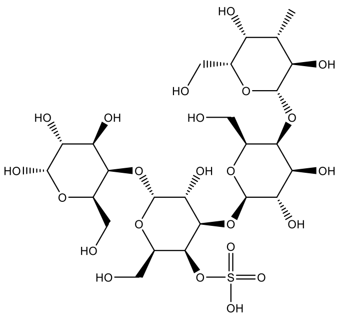

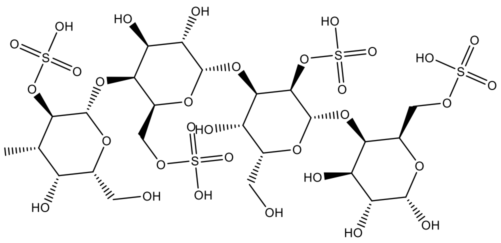

DL-hybrid sulfated galactan

DL-galactan hybrid C2S-3

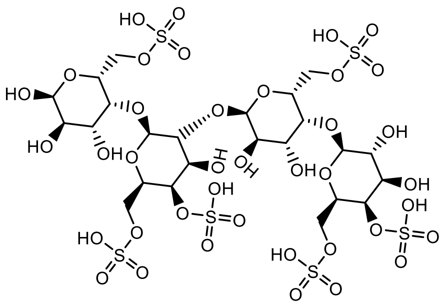

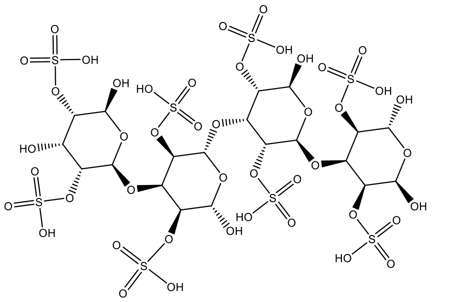

Sulfated galactan

Sulfated mannan

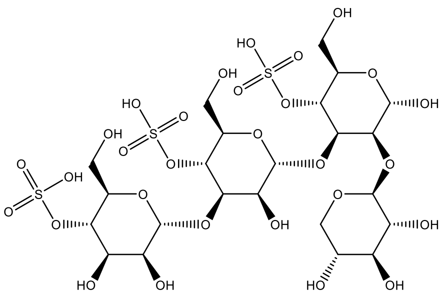

Sulfated xylomannan

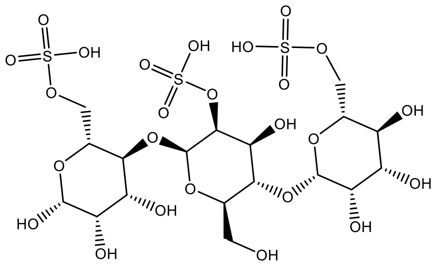

Sulfated xylomannans: α-(1→3)-linked D-mannopyranosyl residues

Green Algae

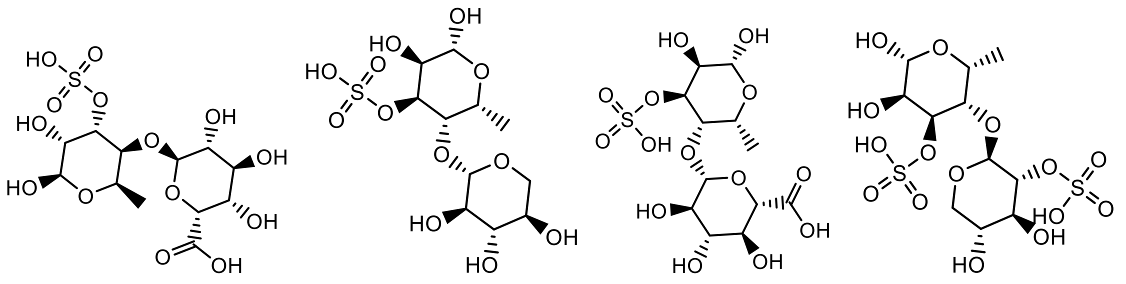

Ulvans

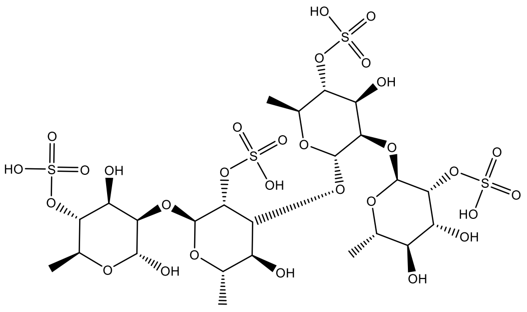

Sulfated heterorhamnan

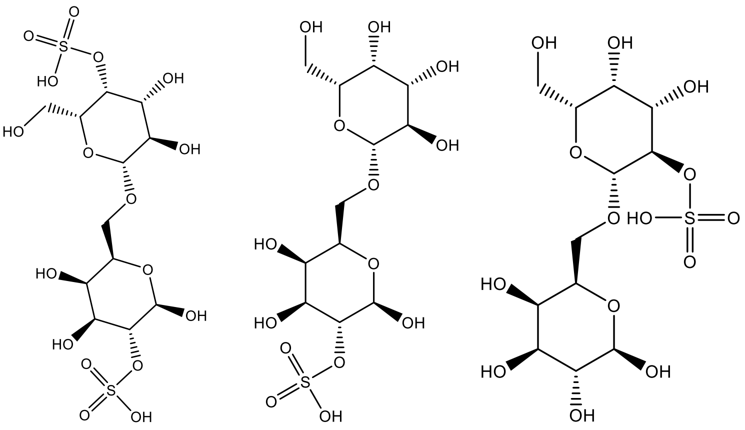

Sulfated arabinoxylogalactans

Blue-Green Algae

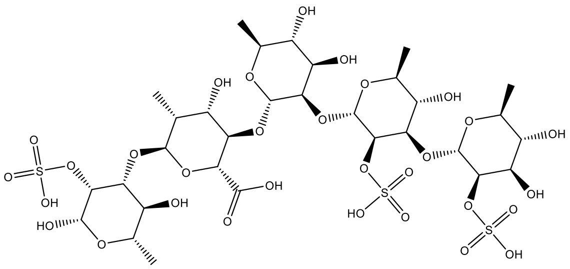

Spirulan (Ca-SP)

Marine animal-derived sulfated polysaccharides

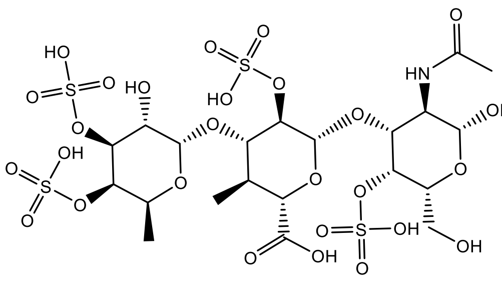

Sea cucumber sulfated polysaccharide (SCSP)

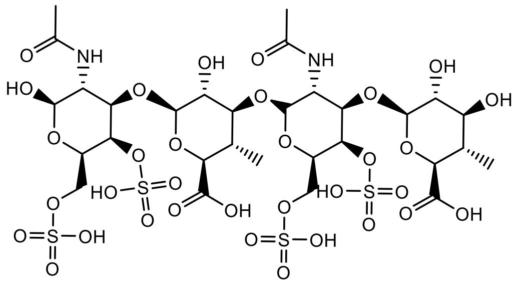

Chondroitin sulphate E (CS-E)
